# Characterization and functional analysis of the proteins Prohibitin 1 and 2 in *Trypanosoma cruzi*

**DOI:** 10.1371/journal.pntd.0009322

**Published:** 2021-04-08

**Authors:** Ana K. Ibarrola-Vannucci, Luis M. De Pablos, Lissette Retana-Moreira, Alberto Cornet-Gomez, Teresa Cruz-Bustos, Alejandro G. Schijman, José L. Ramírez, Susana Vílchez, Antonio Osuna

**Affiliations:** 1 Instituto de Biotecnología. Departamento de Parasitología, Grupo CTS183, Universidad de Granada, Spain; 2 Laboratorio de Biología Molecular de la Enfermedad de Chagas, Instituto de Investigaciones en Biología Molecular e Ingeniería Genética “Dr Héctor Torres” (INGEBI), Consejo Nacional de Ciencia y Tecnología (CONICET), Buenos Aires, Argentina; 3 Biotechnology Center, Instituto de Estudios Avanzados, Instituto de Biología Experimental, Universidad Central de Venezuela, Caracas, Venezuela; 4 Departamento de Bioquímica, Facultad de Ciencias, Universidad de Granada, Spain; University of Texas at El Paso, UNITED STATES

## Abstract

**Background:**

Chagas disease is the third most important neglected tropical disease. There is no vaccine available, and only two drugs are generally prescribed for the treatment, both of which with a wide range of side effects. Our study of *T*. *cruzi* PHBs revealed a pleiotropic function in different stages of the parasite, participating actively in the transformation of the non-infective replicative epimastigote form into metacyclic trypomastigotes and also in the multiplication of intracellular amastigotes.

**Methodology/principal findings:**

To obtain and confirm our results, we applied several tools and techniques such as electron microscopy, immuno-electron microscopy, bioinformatics analysis and molecular biology. We transfected *T*. *cruzi* clones with the PHB genes, in order to overexpress the proteins and performed a CRISPR/Cas9 disruption to obtain partially silenced PHB1 parasites or completely silenced PHB2 parasites. The function of these proteins was also studied in the biology of the parasite, specifically in the transformation rate from non-infective forms to the metacyclic infective forms, and in their capacity of intracellular multiplication.

**Conclusion/significance:**

This research expands our understanding of the functions of PHBs in the life cycle of the parasite. It also highlights the protective role of prohibitins against ROS and reveals that the absence of PHB2 has a lethal effect on the parasite, a fact that could support the consideration of this protein as a possible target for therapeutic action.

## Author summary

*Trypanosoma cruzi* is the etiological agent of American trypanosomiasis or Chagas disease. Among the trypanosomes known to affect humans, this is the only one with an obligatory intracellular multiplication stage in the mammalian host called amastigote. In this work, we describe for the first time the function and location of *Trypanosoma cruzi* proteins Prohibitin 1 and 2. Prohibitins are highly conserved proteins that belong to the Stomatin-Prohibitin-Flotillin-HflC/K (SPFH) protein superfamily and share a SPFH domain. PHBs are ubiquitous and play important roles in many cellular processes. Although they are mainly located in yeast mitochondria, nematodes and humans, PHBs could also be present in the nucleus and in cytosolic membranes. The localization of *T*. *cruzi* PHBs is stage-specific, showing a cytoplasmic membrane distribution in the human infective trypomastigote stages and in the amastigote forms. In the epimastigote form, PHBs bind Fe^3+^ ions, exerting a protective effect against oxidative stress. While the overexpression of PHB1 and PHB2 showed no significant differences in the parasite’s infectivity, it caused an increase in the intracellular multiplication of the amastigote form. The deletion of the PHB2 gene had a lethal effect on the parasite, whereas PHB1 partial silencing caused a significant decrease in ROS detoxification. Together, these results indicate that PHBs could participate in metacyclogenesis in ROS detoxification and could play a role in the replication of the intracellular stage of *T*. *cruzi*. Besides, our results suggest the potential role of PHB2 as a target for drug therapy.

## Introduction

*Trypanosoma cruzi*, a flagellate protozoan parasite that belongs to the order Kinetoplastida, is the etiologic agent of Chagas disease, a neglected tropical disease [1,2] endemic in 21 Latin American countries and distributed from the south of the USA to Argentina and Chile [3]. Due to human movements, e.g. migration and tourism, Chagas disease has a global expansion nowadays, emerging in areas considered a few decades ago as free of the illness, such as the North of the USA, Canada, Europe, Australia or Japan [4]. Despite the importance and severity of the disease, there is neither a truly specific treatment for the chronic phase nor effective prophylaxis for this disease [5].

*T*. *cruzi* has a complex life cycle that involves mammalian and insect hosts. In insects (Hemiptera, Reduviidae), the development of the parasite occurs in the gut. The bloodstream trypomastigote (T) stage is ingested by the insect from an infected mammalian host and differentiates into the epimastigote stage (E), a highly proliferative stage that is non-infective to the mammalian host. After 8–15 days, the E form is transformed into non-proliferative metacyclic trypomastigotes (M) in the terminal intestine and rectum of the insect. This M stage is excreted in the insect’s dejections, together with the feces and urine. When the metacyclic trypomastigotes infect mammalian host cells, they transform into the intracellular amastigote stage (A), a form that multiplies and occupies the cytoplasm of the host cell and, after several rounds of multiplication, differentiates into the non-multiplicative but infective trypomastigote. Upon host cell lysis, the T stage is released into the intercellular space, then into the bloodstream where it can infect other cells (maintaining the mammalian infection) or be ingested by other triatomines, thus completing its life cycle [6].

Prohibitins (PHBs) are highly conserved proteins of the Stomatin-Prohibitin Flotillin-HflC/K (SPFH) protein superfamily and share a SPFH domain, also called PHB or band-7 domain. This domain can be found in eukaryotic cells, bacteria, and archaea [7–11]. Despite its high degree of conservation, the precise function of the SPFH or band-7 domain remains unknown in most organisms [8]. The SPFH protein superfamily includes proteins that are anchored in cellular membranes, the plasma membrane (PM), early endosomes, the Golgi apparatus, mitochondria and the endoplasmic reticulum (ER) [10].

Among protozoan parasites of the order Kinetoplastida, PHB1 has been characterized in *Trypanosoma brucei* [12] and *Leishmania donovani* [13]. Recently, our research group characterized PHB1 and PHB2 in *Leishmania major* [14]. In *T*. *brucei*, PHB1 is located in the mitochondria, forming a high molecular weight complex, and the ablation of PHB1 or both PHB1 and PHB2 led to a decrease in mitochondrial membrane potential and exhibited significant morphological alterations of this organelle [12]. In *L*. *donovani* [13], PHB1 is present on the flagellar surface and in the aflagellar pole, and the overexpression of PHB1 increased the amount of protein in the cell membranes, resulting in parasites with greater capability of infection. Cruz-Bustos et al. [14] found PHB1 in the mitochondria and plasma membrane of the promastigote and amastigote forms of *L*. *major*, whereas PHB2 was located in the nucleus. Moreover, the capability of PHBs to bind iron was demonstrated and it was proposed that these proteins could play a role in the protection of the parasite against ROS. The importance of PHB1 in the biology of *Leishmania infantum* has led to propose this protein as a vaccine candidate for visceral leishmaniosis [15].

As mentioned above, *T*. *cruzi* is exposed to a wide variety of environments to which the parasite needs to adapt and survive and, therefore, applies multiple physiological and biochemical changes; one of these challenges faced is the oxidative stress inside the vertebrate or invertebrate hosts. Considering the known properties of PHBs to act as reactive oxygen species (ROS) chaperones, in the present work we examined the locations of PHB1 and PHB2 inside *T*. *cruzi* and studied their role as oxidative stress protectors. We also determined the effect of the overexpression and suppression of these proteins in the life cycle of this parasite.

## Results

### Bioinformatic analysis of putative PHB1 and PHB2 proteins of *T*. *cruzi*

**Fig 1:**
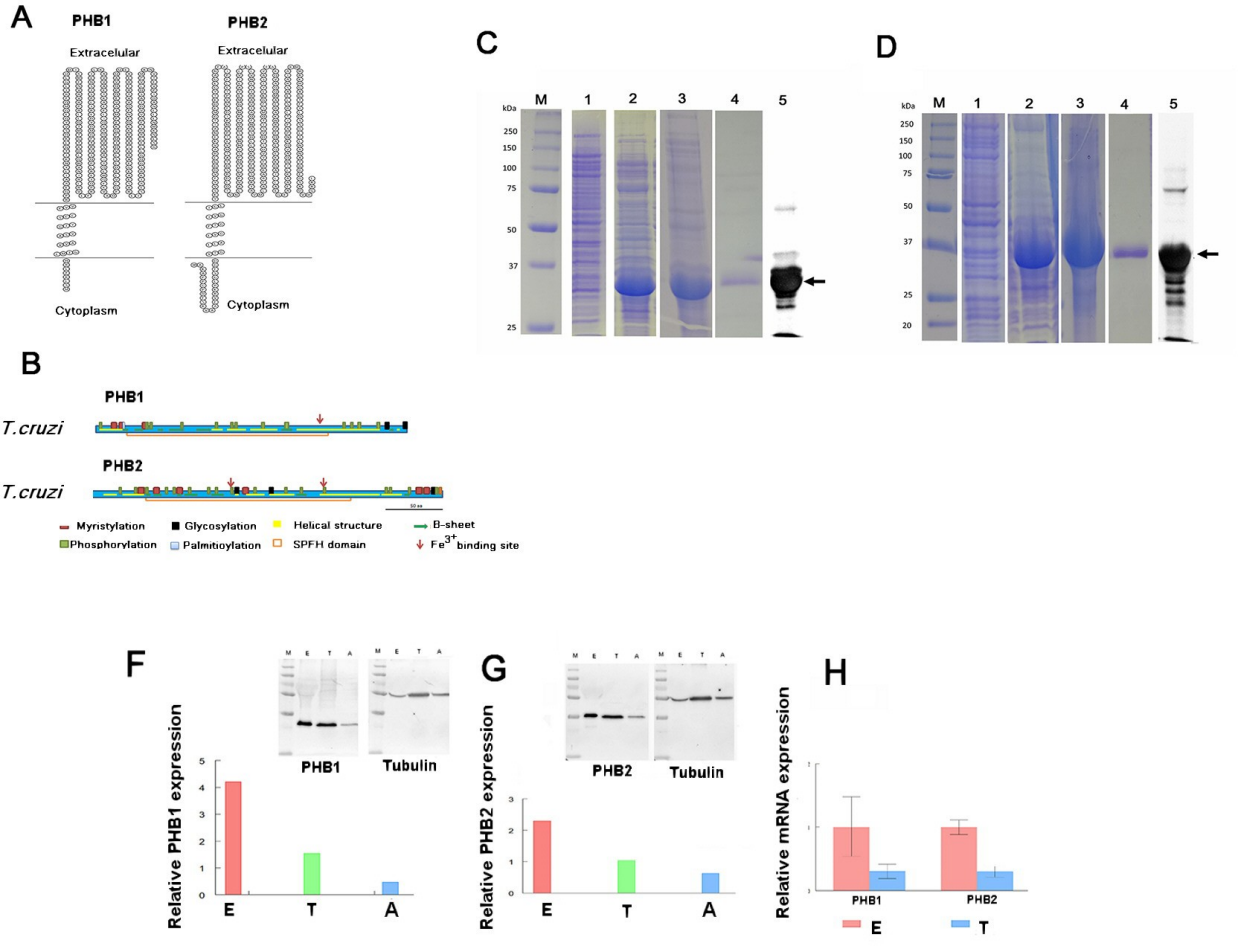
Bioinformatic predictions for PHBs. **(A)** Topological model of PHB1 and PHB2 from *T*. *cruzi*. Both proteins have a transmembrane helix that crosses the lipid bilayer, with their N-terminal domain in the cytosol and their C-terminal in the extracellular space. **(B)** Topology predictions were performed using CCTOP (http://cctop.enzim.ttk.mta.hu/) and TOPO2 transmembrane protein display software (http://www.sacs.ucsf.edu/cgi-bin/open-topo2.py). Prediction of the secondary structure, post-translational modifications, SPFH motif and Fe^3+^ binding sites for PHB1 and PHB2. Expression of *T*. *cruzi* PHB1 **(C)** and PHB2 **(D)** in bacteria, isolation and purification: 1: total proteins of *E*. *coli* transformed with the recombinant plasmids with the *phb* gene without IPTG induction; 2: protein profile after induction with IPTG: 3: sample obtained from solubilized inclusion bodies; 4: sample after purification from SDS-PAGE, 5: recognition by Western blot with the primary antibody against the His-tag. Relative expression of PHB1 **(F)** and PHB2 **(G)** in the three different stages of the *T*. *cruzi* DM28c strain. Calculations were made by measuring the intensity of the bands obtained in the Western Blot assays (E epimastigote forms; T, trypomastigote forms and A amastigote forms). Lanes: M: All Blue molecular weight marker (Bio-Rad); E: total epimastigote proteins; T: total trypomastigote proteins; A: total amastigote proteins. **(H)** Relative mRNA quantification of the expression of the *phb1* and *phb2* genes by RT-qPCR in epimastigotes (E) and blood stream trypomastigotes (T). Expression levels were normalized with the *gapdh* expression for each stage.

Two homologous ORFs of 819 and 921 bp were identified as putative *phb1* and *phb2* genes in the *T*. *cruzi* genome, respectively. The *phb1* ORF coded for a putative protein of 30.9 kDa. The specialized signal prediction software SignalBlast and SignalIP 4.1 failed to detect any signal peptide for this protein. The analysis of the amino acid sequence of the putative PHB1 with CCTOP software showed a transmembrane segment between amino acids 9–27, and the analysis using the TOPO2 software showed that it was integral membrane protein that crosses the lipid bilayer, leaving its N-terminal domain in the cytosol and the C-terminal domain outside the cell (Fig 1A). This prediction was confirmed using the Interpro database, where the protein was described as a component of the cell membrane (GO: 0016020).

The *phb2* ORF coded for a 33.2 kDa protein and the analysis of its amino acid sequence with CCTOP also showed a transmembrane segment between the amino acids 27–44, with the N-terminal end localized in the cytoplasm and the C-terminal end outside the cell membrane (Fig 1A). Similarly, Interpro determined that the putative PHB2 is a cellular component of cell membranes (GO: 0016020).

Several Fe^3+^ ligand binding sites were predicted in both putative proteins (Fig 1B). PHB1 showed a binding site at residues E199 and Q202 (predicted with IonCom), whereas PHB2 showed two possible binding motifs for Fe^3+^ (named EXXE) at the positions E222-E225 and E134-E137, located near phosphorylation sites (predicted with Galaxy 39).

Sequence comparisons of the PHBs of *T*. *cruzi* with their orthologues in *T*. *brucei*, *L*. *major*, *L*. *donovani* and *Homo sapiens* showed 84% and 83% identity with PHB1 and PHB2 of *T*. *brucei*, ~81% and 74% identity with PHB1 and PHB2 of *Leishmania* and 48% and 47% identity with PHB1 and PHB2 of *H*. *sapiens* (S1A and S1B Fig). The SPFH/PHB/Band 7 common domain in the proteins that belong to the SPFH superfamily was also detected (red line in the supplementary S1 Fig alignment).

### Expression and purification of PHB1 and PHB2 recombinant proteins

*T*. *cruzi’*s *phb1* and *phb2* ORFs were cloned in the expression vector pQE30-Xa as described in Materials and Methods. The resulting clones, namely *Escherichia coli* pQEHB1 and pQEHB2, were tested in a time-course experiment for protein expression. Two very intense bands of 33 and 35 kDa were observed in IPTG-induced cultures at 0 and 4 h (Fig 1C and 1D, marked as 1 and 2).

As PHB1 and PHB2 proteins were mostly present in inclusion bodies in the insoluble fraction of the culture (Fig 1C and 1D, marked as 3), it was necessary to solubilize them in a denaturing solution as described in Materials and Methods, and further purify them by excision and elution of the bands from polyacrylamide gels for the immunization experiments (Fig 1C and 1D, marked as 4). Protein bands were detected by Western blot using the anti-PoliHisTag antibody (Fig 1C and 1D, marked as 5).

After verifying the specificity and titers of the obtained antibodies, these were employed for detecting PHB1 and PHB2 in lysates of different stages of *T*. *cruzi* DM28c strain. Single bands with the expected sizes of 31 kDa for PHB1 and 33 kDa for PHB2 were detected (Fig 1F and 1G). Besides, these antibodies did not recognize other proteins of the SPFH superfamily that share the common domain SPFH/PHB. Antibodies produced using the recombinant PHB1 also recognized a single band in the promastigote forms of *L*. *major* and two bands in the procyclic trypomastigote forms of *T*. *brucei*, but did not recognize the PHB1 of Vero cells (S2A Fig). The antibody against recombinant PHB2 showed a higher specificity, cross reacting only with the *T*. *brucei* extract, but not with the extracts of *Leishmania* or Vero cells (S2B Fig).

Protein expression studies in the different stages of *T*. *cruzi* were performed using Western blot. The expression levels of both PHBs were higher in the epimastigote stage when compared to the trypomastigote and the amastigote forms (Fig 1F and 1G). These results paralleled those of RT-qPCR (Fig 1H) obtained by quantifying the mRNA levels; thus, the transcription of the *phb* genes in *T*. *cruzi* could be related to the protein expression. The expression levels of PHB1 and PHB2 were also compared among the epimastigote forms of *T*. *cruzi* stocks from different DTUs (S3 Fig). The highest expression levels for both PHB1 and PHB2 proteins were observed in the Y strain (DTU II); however, differences were statistically significant only in the case of PHB2.

### Immuno-localization of PHB1 and PHB2 proteins in *T*. *cruzi*

**Fig 2:**
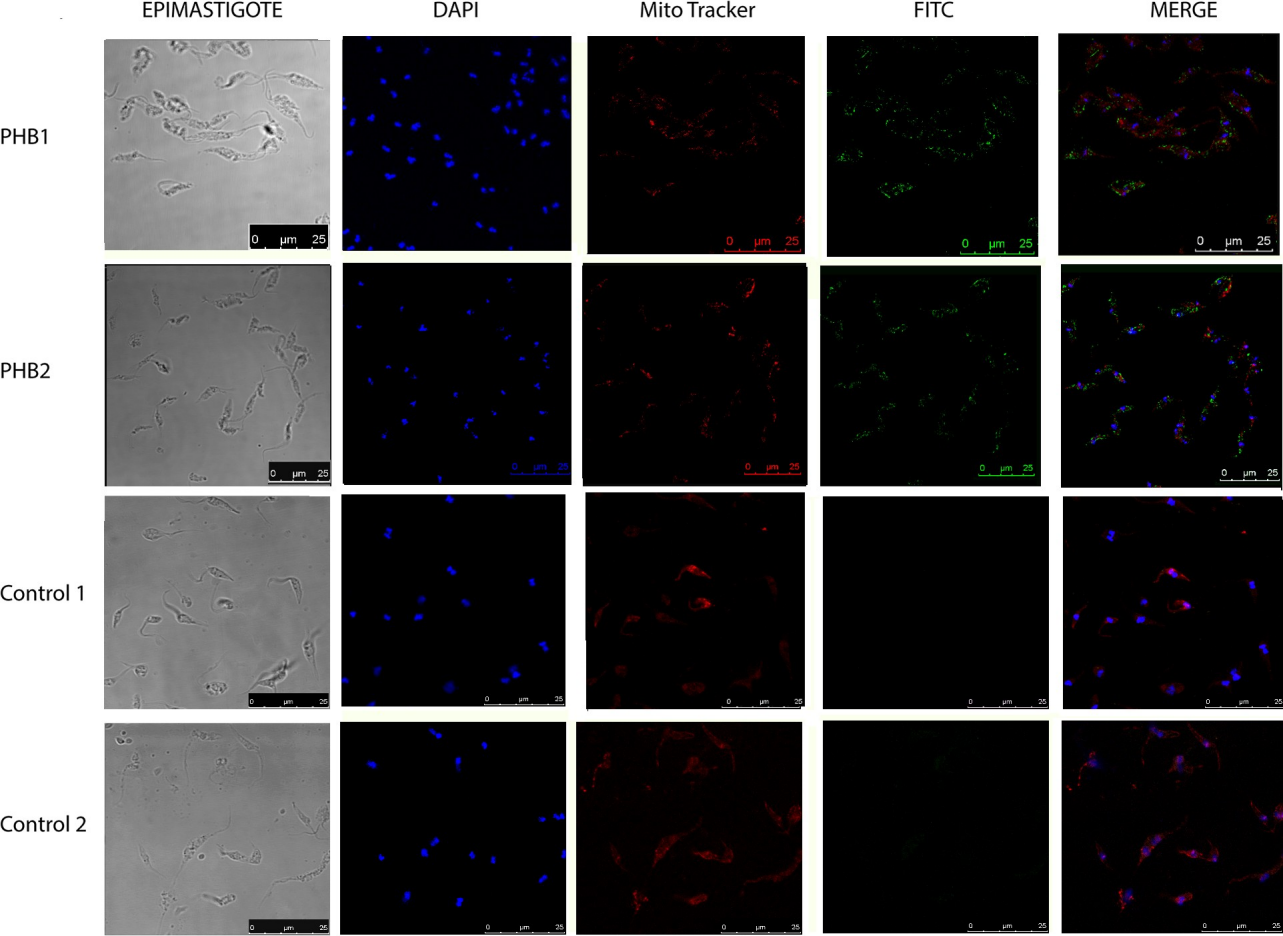
Fluorescence microscopy analysis in epimastigote forms of PHB1 and PHB2 using antibodies produced against *T*. *cruzi* PHB recombinant proteins. In blue, DAPI-labeled DNA; in red, the mitochondria labeled with MitoTracker; in green, PHB1 and PHB2 detected with FITC-conjugated secondary antibody. The merged images show the overlap of the green, red and blue channels.

**Fig 3:**
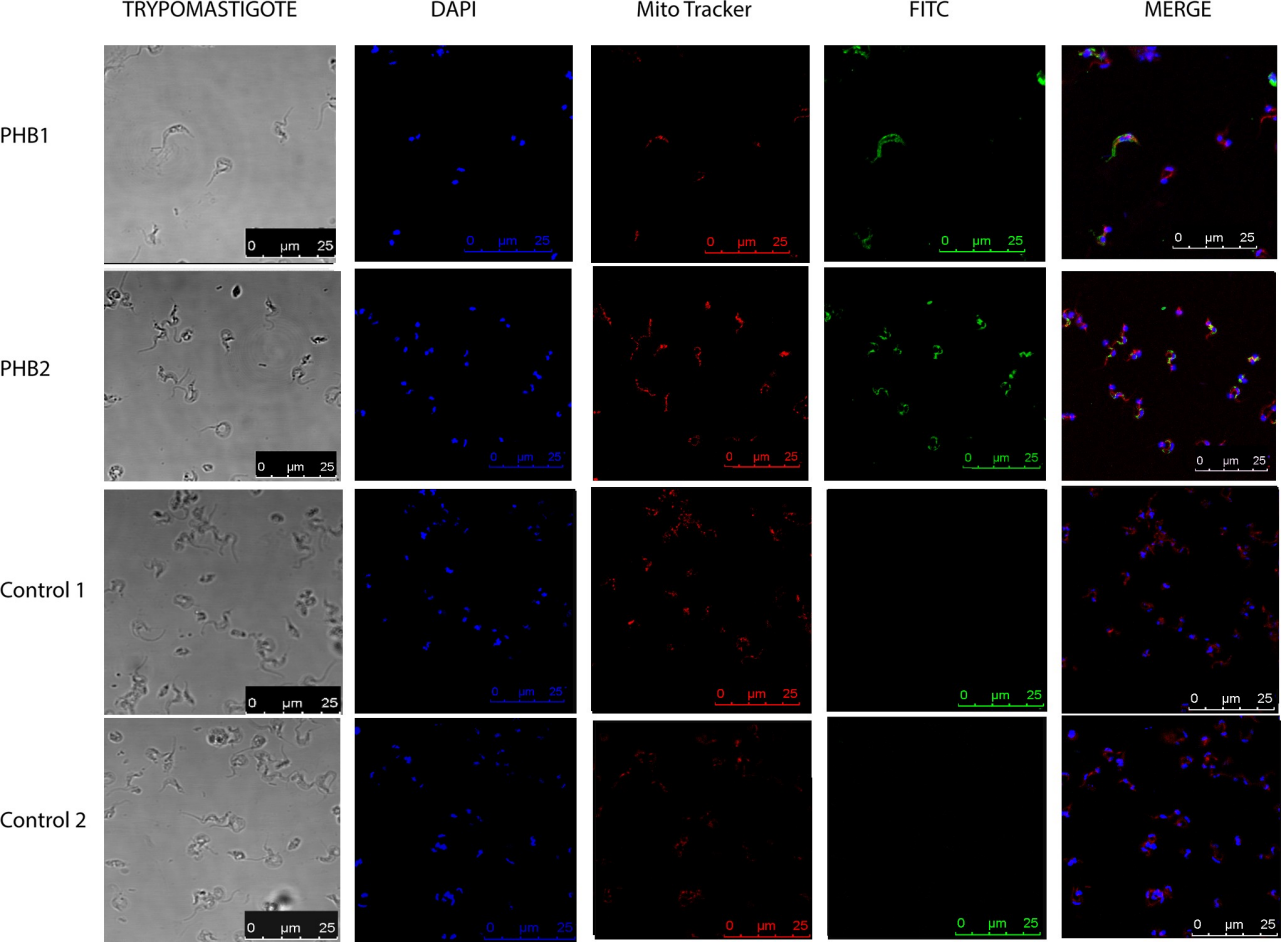
Fluorescence microscopy analysis in trypomastigote forms of PHB1 and PHB2 using antibodies produced against *T*. *cruzi* PHB recombinant proteins. In blue, DAPI-labeled DNA; in red, the mitochondria labeled with MitoTracker; in green, PHB1 and PHB2 detected with FITC-conjugated secondary antibody. The merged images show the overlap of the green, red and blue channels.

**Fig 4:**
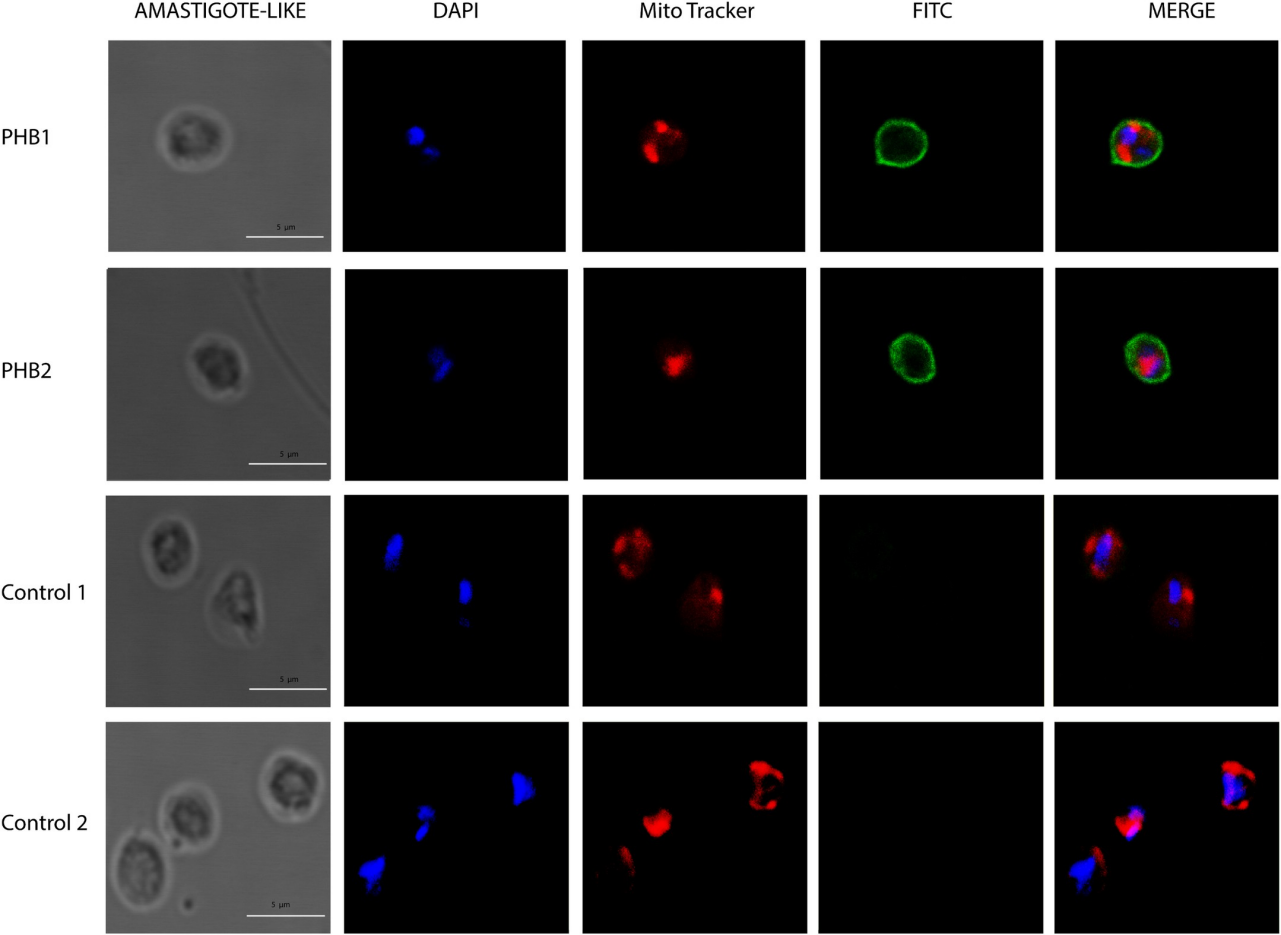
Fluorescence microscopy analysis in amastigote forms of PHB1 and PHB2 using antibodies produced against *T*. *cruzi* PHB recombinant proteins. In blue, DAPI-labeled DNA; in red, the mitochondria labeled with MitoTracker; in green, PHB1 and PHB2 detected with FITC-conjugated secondary antibody. The merged images show the overlap of the green, red and blue channels.

The localization of PHB1 and PHB2 was carried out in the epimastigote, trypomastigote, and amastigote stages of *T*. *cruzi* Dm28c strain using laser confocal microscopy and the Mitotracker stain as a mitochondrial marker. Fig 2 shows the immunolocalization of both PHBs in epimastigotes. Although PHB1 is more dispersedly distributed than PHB2, both proteins clustered in multiple locations inside the parasite. PHB1 appears clustered next to the kinetoplast and the posterior cell apical region, while PHB2 is mainly observed next to the kinetoplast, near the nucleus, and at the flagellar pole. A mitochondrial co-localization was also observed for both proteins. On the other hand, in trypomastigotes (Fig 3) and amastigotes (Fig 4), PHB1 and PHB2 were restricted to the parasite´s surface.

**Fig 5:**
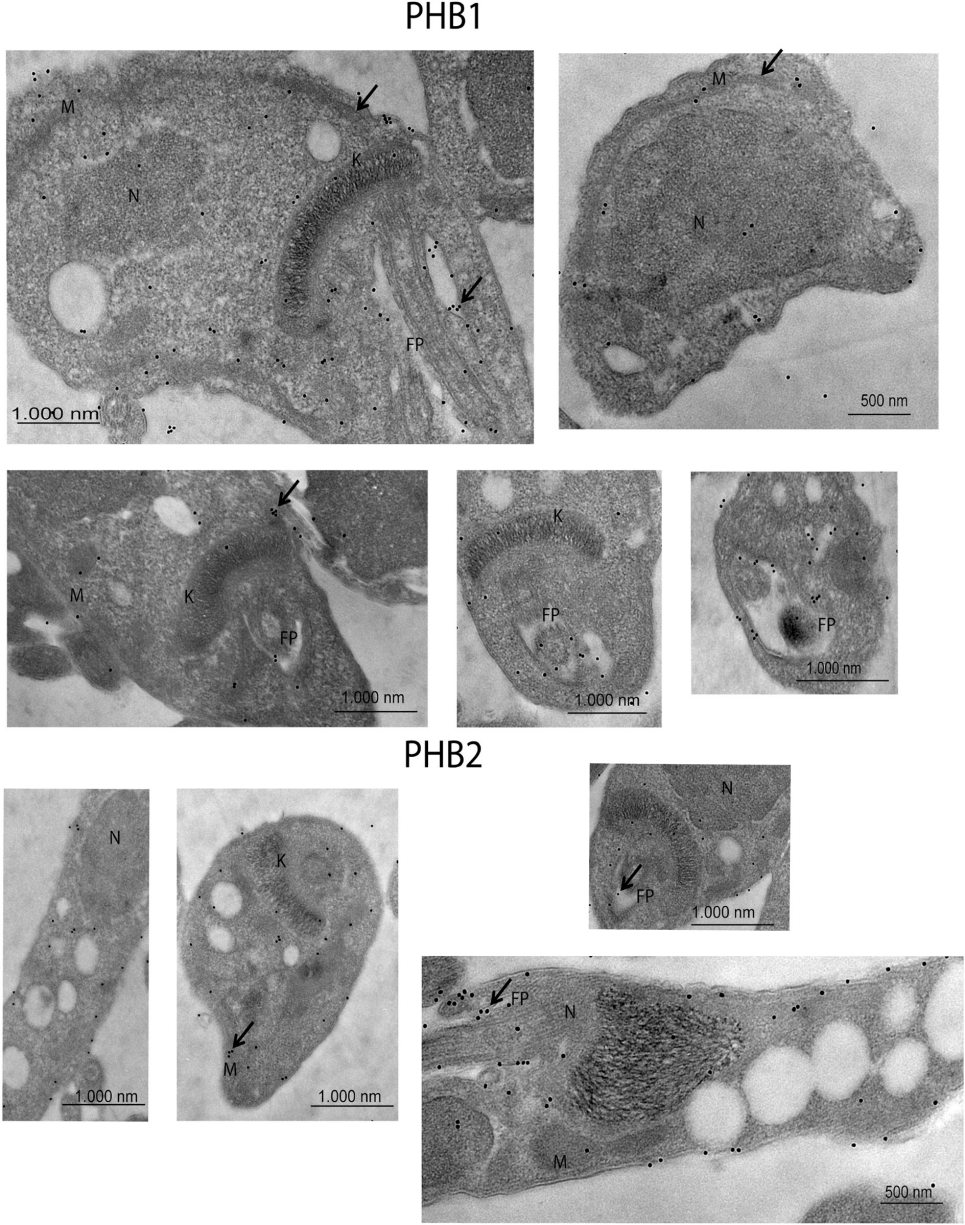
Immunolocalization of PHB1 and PHB2 in epimastigote forms of *T*. *cruzi* by electron microscopy. Ultrathin sections were incubated with anti-PHB1 and anti-PHB2 antibodies, followed by secondary antibodies coupled to 10 nm or/and 18 nm gold particles. Images of control sera are shown in S4 Fig samples incubated with pre-immune sera; control 2: samples incubated only with the secondary antibody. Representative images are shown. K: kinetoplast, M: mitochondria, N: nucleus, FP: flagellar pocket, The control images using the preimmune sera or from non-immunized animals are shown in S4 Fig.

**Fig 6:**
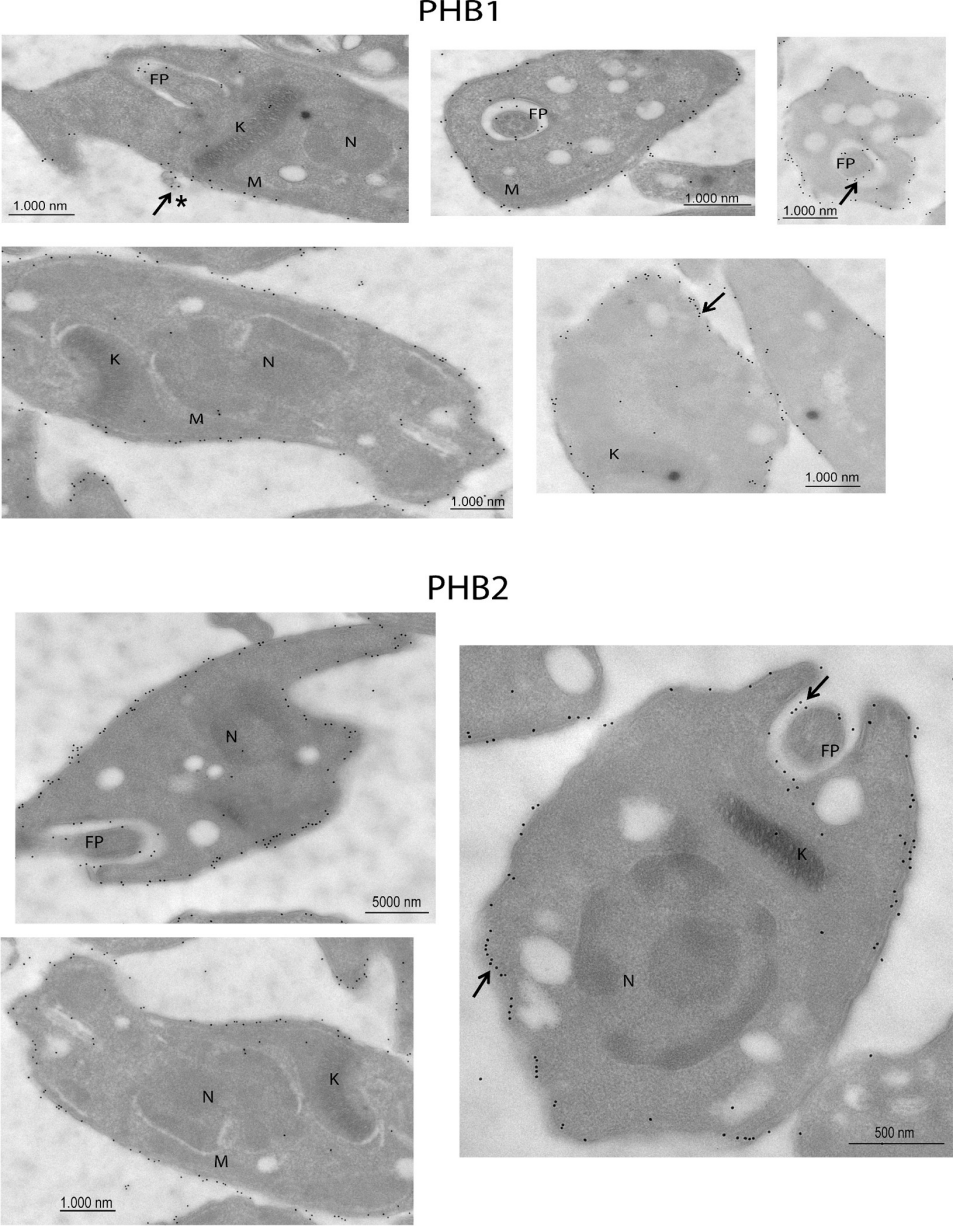
Immunolocalization of PHB1 and PHB2 in trypomastigote forms of *T*. *cruzi* by electron microscopy. Ultrathin sections were incubated with anti-PHB1 and anti-PHB2 antibodies, followed by secondary antibodies coupled to 10 nm or/and 18 nm gold particles. Images of control sera are shown in S4 Fig samples incubated with pre-immune sera; control 2: samples incubated only with the secondary antibody. Representative images are shown. K: kinetoplast, M: mitochondria, N: nucleus, FP: flagellar pocket, *: Extracellular vesicles.

**Fig 7:**
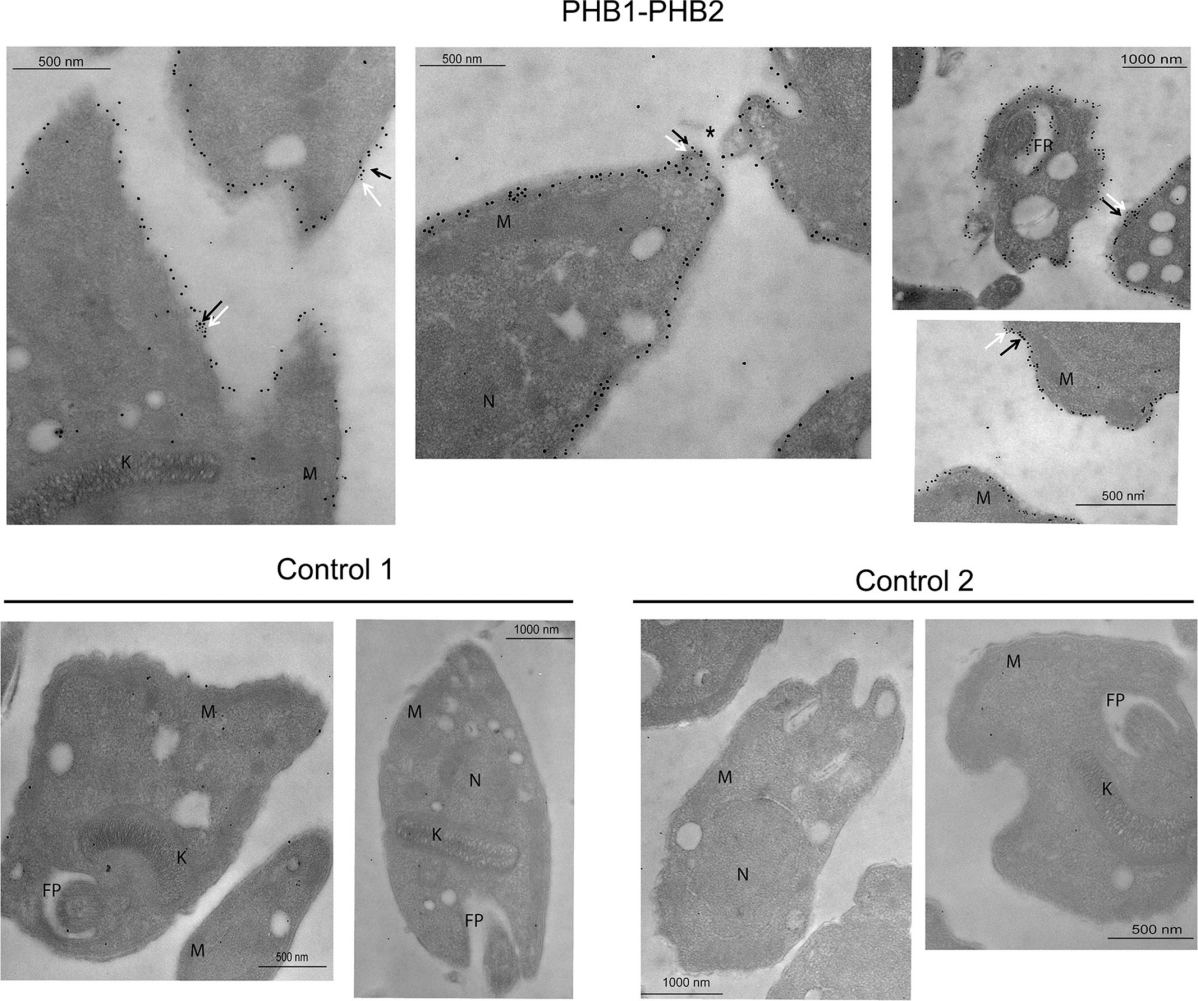
Localization of both proteins, PHB1 and PHB2, close to each other in the plasma membrane of trypomastigotes. Ultrathin sections were incubated with anti-PHB1 and anti-PHB2 antibodies, followed by secondary antibodies coupled to 10 nm or/and 18 nm gold particles. K: kinetoplast, M: mitochondria, N: nucleus, FP: flagellar pocket.

The localization by immunofluorescence was also confirmed by transmission electron microscopy using conjugated colloidal gold antibodies. Thereby, in the epimastigote stage (Fig 5), PHB1 was mainly present at the flagellar pocket, mitochondria and near the kinetoplast, while PHB2 was predominantly located in the flagellar pocket, mitochondria and in the cytoplasmic membrane. Regarding trypomastigotes, (Fig 6) PHB1 and PHB2 were mainly restricted to the parasite´s surface. Additionally, gold particles were also observed in the flagellar pocket and in the extracellular vesicles secreted by the parasite. The immune co-localization of PHB1 (18 nm gold particles) and PHB2 (10 nm gold particles) in trypomastigotes showed that both proteins were, in some cases very, close to each other (Fig 7). Control of negative and preimmune sera is shown in S4 Fig. As control of the specificity of the antibodies to the transfected epimastigotes, Protein A labelled gold particles were employed to localize the Fc present in the TAPTag, since the transfection vector has a Fc domain expressed together with the protein, with affinity to Protein A (S7 Fig).

### PHBs protect DNA from iron-induced oxidative damage

Ion ligand-binding sites for Fe^3+^ were predicted *in silico* for both PHB1 and PHB2 proteins. The affinity for Fe^3+^ ions was further verified experimentally with the purification of the native PHBs by immobilized metal ion affinity chromatography (IMAC). S5 Fig shows the Western blot results for the detection of PHB1 and PHB2 after their purification using the Fe^3+^ affinity columns. These results confirmed that both proteins were able to bind these ions.

After confirming the iron-binding ability of the PHBs, it was assessed whether these proteins were able to protect DNA from the oxidative iron damage as described for *L*. *major*. To test this hypothesis, a supercoil strand break assay was performed.

**Fig 8:**
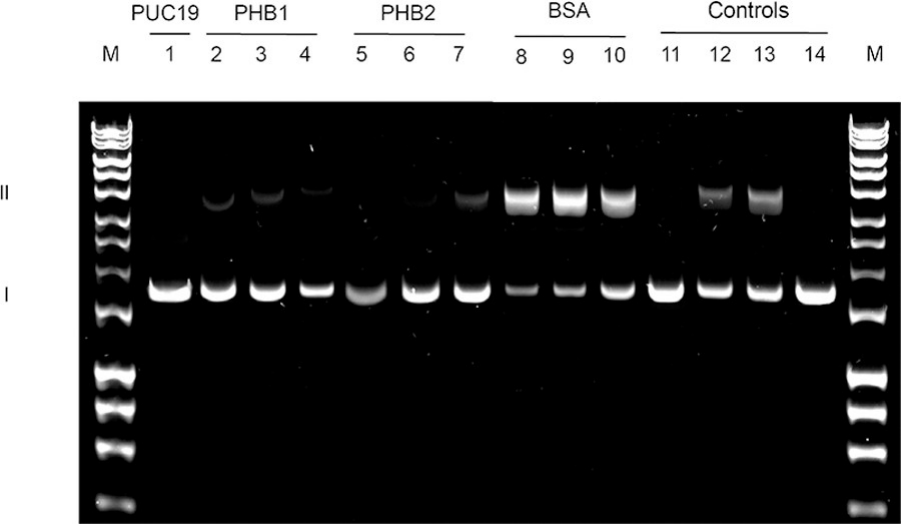
Supercoil DNA relaxation assay and ROS quantification assays by flow-cytometry. Agarose gel electrophoresis of pUC19 DNA after being exposed to ROS. Lane 1: DNA; lanes 2–4: DNA plus 50 μM FeCl_3_, 10 mM H_2_O_2_ and decreasing PHB1 concentrations of 500, 250 and 125 μg/mL, respectively. Lanes 5–7: DNA plus 50 μM FeCl_3_, 10 mM H_2_O_2,_ and decreasing PHB2 concentrations of 500, 250 and 125 μg/mL, respectively. In addition to pUC19 DNA, the controls employed contained the following components: lanes 8–10: DNA plus 50 μM FeCl_3_, 10 mM H_2_O_2,_ and a decreasing BSA concentration (500, 250 and 125 μg/ mL), respectively; lane 11: only FeCl_3_; lane 12: only H_2_O_2_; lane 13: the mixture of FeCl_3_ and H_2_O_2_; lane 14: the mixture of PHB1 and PHB2.

The effect of ROS generated by H_2_O_2_ and FeCl_3_ over the circular supercoiled plasmid pUC19 DNA is shown in Fig 8. Both PHB recombinant proteins protected DNA from nicking, thus preserving the DNA in its supercoiled configuration. However, PHB2 showed a greater protective effect than the PHB1 recombinant protein. BSA used instead of the PHBs failed to protect the DNA, as revealed by the increase of relaxed circular DNA. As expected, DNA degradation was also observed in the experiments using FeCl_3_ and H_2_O_2_.

### Effect of the overexpression of PHBs on the growth, differentiation and infectivity of *T*. *cruzi*

**Fig 9:**
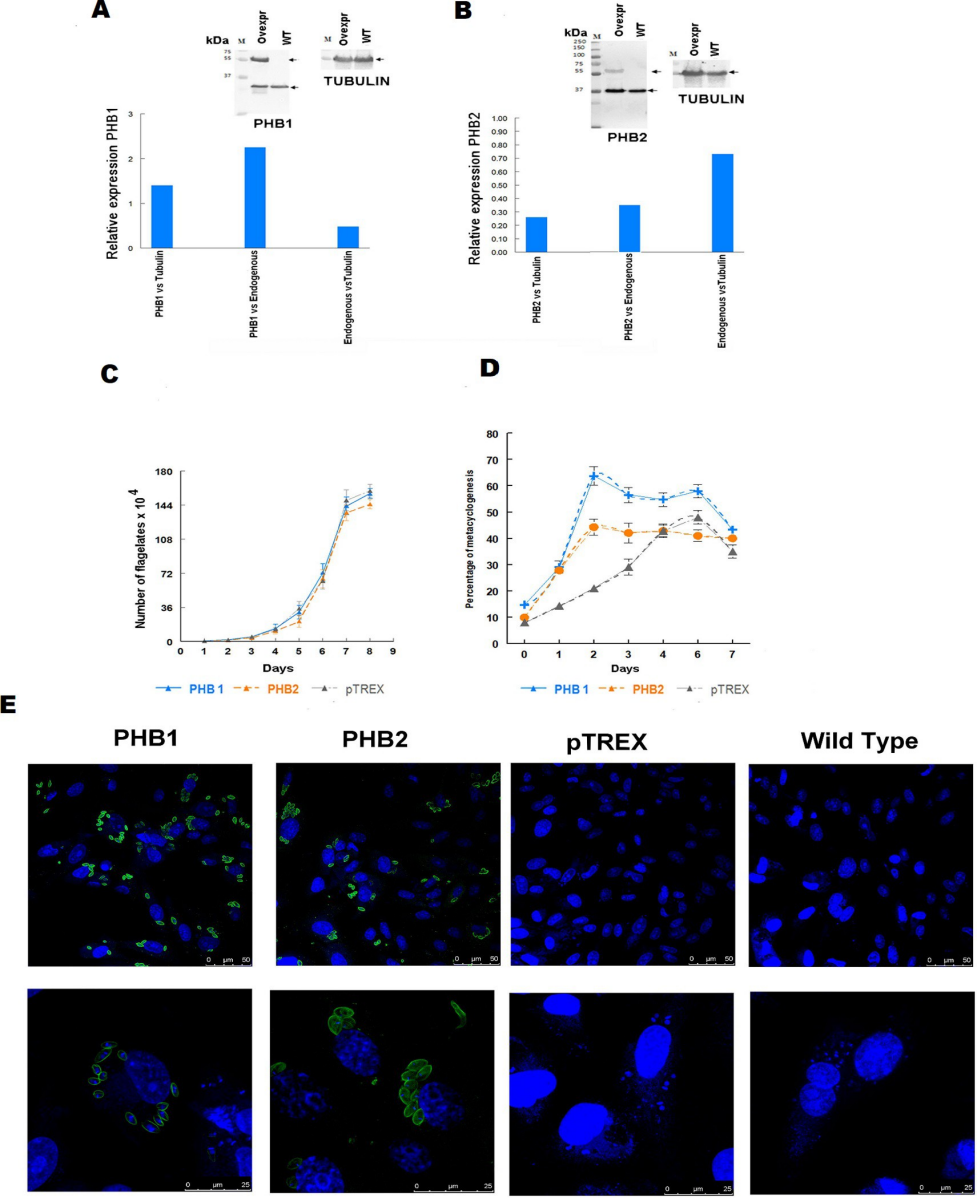
Overexpression of PHBs in different stages of *T*. *cruzi*. **(A)** Relative expression of recombinant PHB1 versus the endogenous PHB1 and tubulin in the epimastigote forms. **(B)** Relative expression of recombinant PHB2 versus the endogenous PHB2 and tubulin in the epimastigote forms. **(C)** Eight-day growth curves of the clones: *T*. *cruzi* (pTAPHB1) overexpressing the recombinant PHB1, *T*. *cruzi* (pTAPHB2) overexpressing the recombinant PHB2, and the control *T*. *cruzi* (pTAP). **(D)** Differentiation kinetics of epimastigotes into metacyclic trypomastigote forms of PHB recombinant cell lines. **(E)** Confocal microscopy of Vero cells 72 h post-infection with trypomastigotes of the recombinant cell lines. Anti-PHB1 and anti-PHB2 antibodies were used as the primary antibodies, and FITC anti-rat IgG antibodies (Sigma, Madrid, Spain) were employed as secondary antibodies. DAPI dye (blue) was employed as the DNA marker.

The clonal cell lines of *T*. *cruzi* overexpressing the PHB1 and PHB2 proteins were generated using the integrative vector pTREXTAPtag-GW carrying the *phb* genes (*T*. *cruzi* (pTAPHB1) and *T*. *cruzi* (pTAPHB2)) with the objective of analyzing the phenotypic effects of the overexpression of both proteins in the biology of the parasite. First, the TAPtag was immunodetected in the crude extracts of *T*. *cruzi* (pTAPHB1) and *T*. *cruzi* (pTAPHB2) (S6 Fig). Moreover, the fused proteins showed the expected sizes (57 kDa for PHB1 and 60 kDa for PHB2), which indicates that both proteins were successfully expressed in the parasite. The results of the overexpression of these proteins are shown in Fig 9A and 9B.

Growth curves of *T*. *cruzi* (pTAPHB1) and *T*. *cruzi* (pTAPHB2) in the epimastigote stages showed that the overexpression of PHB1 or PHB2 did not affect the growth of this stage with respect to the wild-type (Fig 9C). However, a clear effect was observed on the metacyclogenesis process, since it was favored in the clones overexpressing the proteins (Fig 9D). The highest number of metacyclic trypomastigotes was obtained after 48 h of culture in both *T*. *cruzi* (pTAPHB1) and *T*. *cruzi* (pTAPHB2). The percentage of differentiation after 48 h was 64 ± 3.58% in *T*. *cruzi* (pTAPHB1) and 44 ± 3.5% in *T*. *cruzi* (pTAPHB2), whereas the control showed 21± 2.03% even after 72 h.

**Fig 10:**
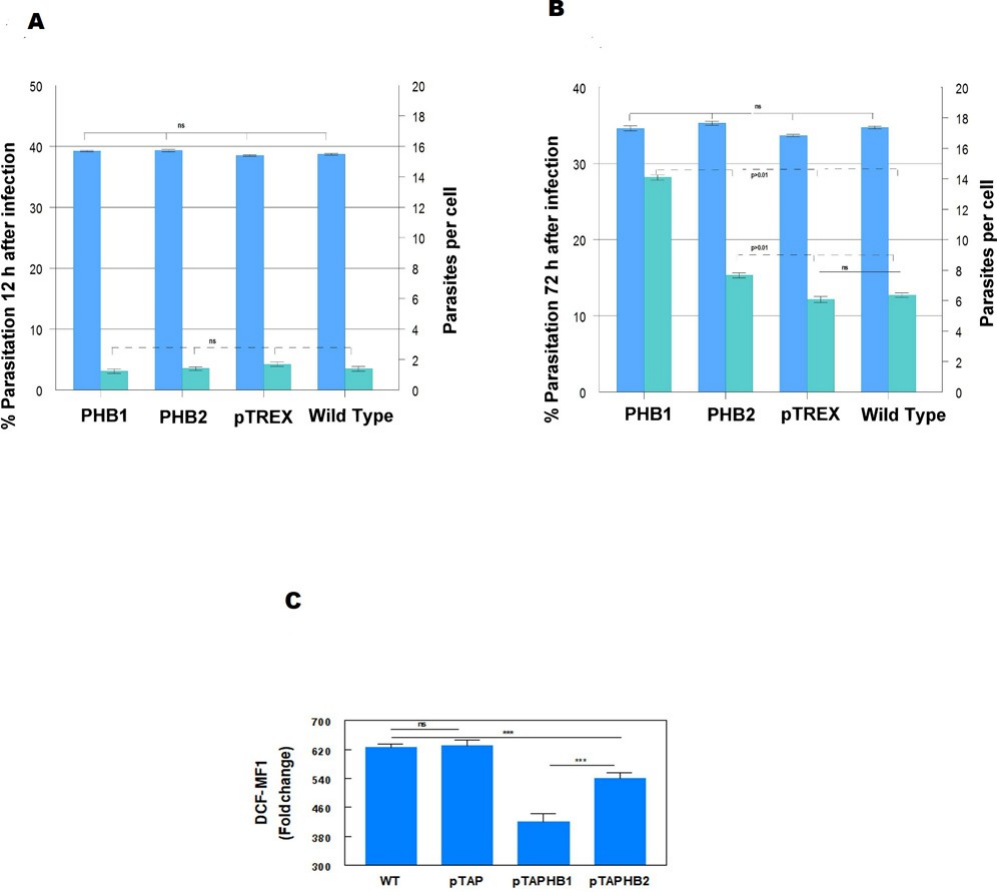
Overexpression of PHBs in different stages of *T*. *cruzi*. **(A)** Percentages of infection and percentage of infected Vero cells 12h post-infection with *T*. *cruzi* trypomastigotes: *T*. *cruzi* (pTAPHB1), *T*. *cruzi* (pTAPHB2), *T*. *cruzi* (pTAP) and *T*. *cruzi* wild-type. (**B**). Percentages of infection and percentage of infected Vero cells after 72 h with *T*. *cruzi* trypomastigotes: *T*. *cruzi* (pTAPHB1), *T*. *cruzi* (pTAPHB2), *T*. *cruzi* (pTAP) and *T*. *cruzi* wild-type. The percentages of infection and the number of amastigotes per cell were obtained by counting the infected cells and the number of amastigotes inside each cell. **(C)** Flow cytometry results of the effect of the H_2_O_2_ treatment over the different parasite cell lines. I: measurements obtained comparing the fluorescence of the treated versus non-treated parasites. II: Ratio of the mean fluorescence intensity (MIF) of the treated versus the untreated samples. The Fisher LSD test p < 0.05 was applied to assess significant differences between the different parasite lines.

Vero cell cultures were infected with the trypomastigote forms of *T*. *cruzi* (pTAPHB), *T*. *cruzi* (pTAPHB2), *T*. *cruzi* (pTREX) and the wild-type (WT) and Fig 9E shows some images of the evolution of the parasites inside the cells after 72 h of infection. At 12 hours postinfection, non-statistically significant differences were observed in the percentages of infection and parasitization of the cells infected with the parasites overexpressing PHB1, PHB2 or the pTAP empty vector, compared to the results obtained using the wild strain (Fig 10A). However, after 72 h of infection (Fig 10B), the parasitization index (number of amastigotes/cell) was significantly higher (p ≤ 0.05) in cells infected with parasites overexpressing PHB1 or PHB2. The parasites overexpressing PHB1 provoked a slightly greater increase in the parasitization index (number of amastigotes per cell) than those overexpressing PHB2.

We subjected *T*. *cruzi* cells overexpressing PHB1, PHB2 and the pTAP control to oxidative stress conditions by adding H_2_O_2_ to the cultures and labeling them with DCFDA, a fluorochrome employed to determine intracellular oxidative stress. As shown in Fig 10C, the overexpressing clones were more resistant to the oxidative stress induced by the H_2_O_2_, suggesting that PHB1 and PHB2 could have a protective role against ROS in *T*. *cruzi*.

### CRISPR/Cas9 disruption

As described in Materials and Methods, the plasmid pTREX/Cas9 was modified with two different sgRNAs specific for the *phb* genes. Donor DNAs were designed to contain the *phbs* 5’ and 3’ UTR regions flanking the gene for blasticidin resistance. Although the donor DNA for *phb2* replacement was successfully constructed, we failed to obtain the one for *phb1*. The 3’ UTR for this gene could not be amplified by PCR even after using three different sets of primers. For this reason, we opted for a simple Cas9 disruption of *phb1*.

The resulting parasite cells, named *T*. *cruzi* (1sgCas9PHB1), *T*. *cruzi* (2sgCas9PHB1), *T*. *cruzi* (1sgCas9PHB2), *T*. *cruzi* (2sgCas9PHB2) and *T*. *cruzi* (ScCas9) harboring the scrambled sgRNA (control) were further characterized.

**Fig 11:**
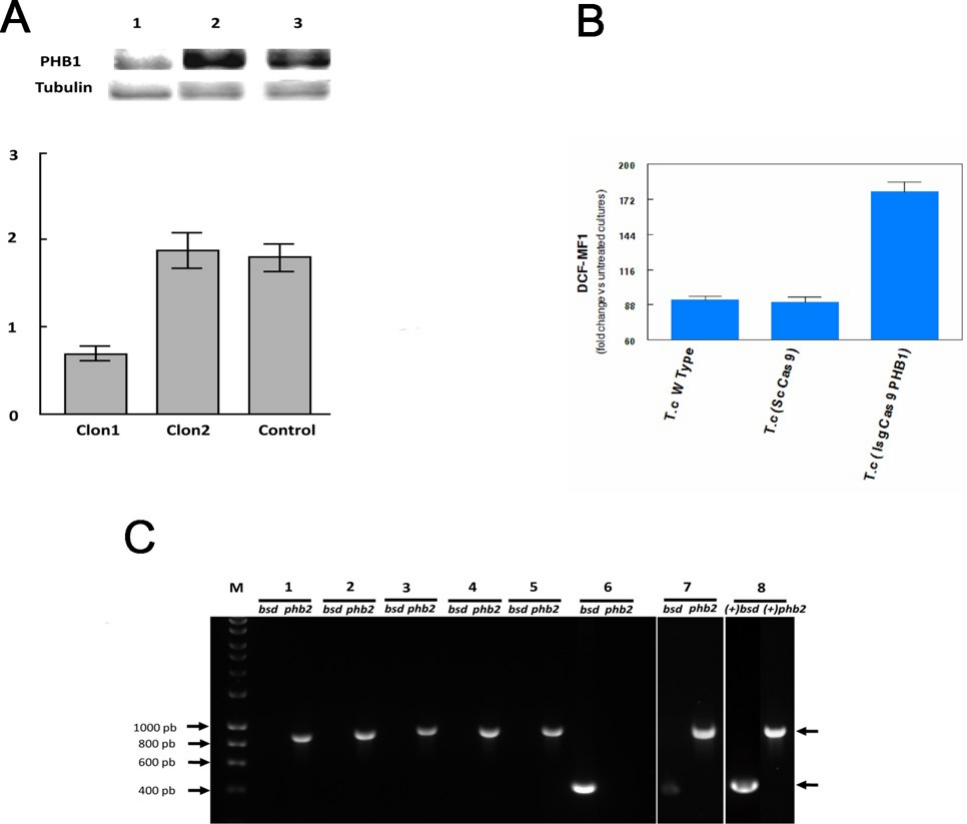
CRISPR/Cas9 disruption. **(A)** Relative expression of PHB1 in two different clones of epimastigotes of *T*. *cruzi* (1sgCas9PHB1), compared to the tubulin control. The relative expression calculations were performed using the area and intensity of the bands obtained (Quantiscan V.1.25) by Western blots. 1,2: different clones of *T*. *cruzi* (1sgCas9PHB1); 3: *T*. *cruzi* (ScCas9) (control). **(B)** Flow cytometry analysis of the parasites *T*. *cruzi* (1sgCas9PHB1) with a decreased PHB1 expression and the control *T*. *cruzi* (ScCas9) strain. I: measurements of DCF for the treated with H_2_O_2_ and untreated samples for each cell line. The Fisher LSD test was employed to access the possible statistical differences (*p* <0.05). **(C)** 1% Agarose gel showing the amplification product obtained after the replacement of the *phb2* gene and the incorporation of the linear fragment FRL2. M: molecular weight marker HyperLadder I (Bioline), 1–7: different clones obtained after the amplification of the *phb2* and blasticidine *bsd* genes; 8: PCR positive controls.

The cell lines in which *phb1* was disrupted (*T*. *cruzi* (1sgCas9PHB1) and *T*. *cruzi* (2sgCas9PHB1)) showed the same growth rates than the parasites transformed with the scrambled sgRNA. After analyzing the PHB1 expression of several clones from these cell lines by Western blot, we selected a clone that showed a reduction in PHB1 protein expression compared to the control (Fig 11A). The integrity of the locus was analyzed by PCR showing no rearrangements of this particular locus (S8 Fig and S1 Table). This clone and the control were treated with H_2_O_2_ and analyzed by flow cytometry using DCFC, as previously described. The results showed that the clone with lower PHB1 expression had a two-fold increase in fluorescence, suggesting that they were less resistant to oxidative stress than the control cells (Fig 11B). This result is consistent with the previous observation that the overexpression of PHB1 reduced the impact of oxidative stress in the cell.

In the case of *phb2*, each sgRNA/pTREX/Cas9 construct was co-transfected with the donor DNA containing the 5´and 3´UTR regions of *phb2* flanking the *bsd* gene. After antibiotic selection, the presence of the *bsd* gene and the absence of the *phb2* gene were confirmed by PCR in the two clones obtained (*T*. *cruzi* (1sgCas9PHB2), *T*. *cruzi* (2sgCas9PHB2)) (Fig 11C). However, after one week, the parasites started to die and the transfected flagellates could only be collected for an ultrastructural analysis before the parasite’s death. TEM studies showed that transfected parasites lacking the PHB2 protein presented a different morphology of the mitochondrial membrane and large cytoplasmic vacuolization, suggesting that the absence of PHB2 has a lethal effect on the parasite.

## Discussion

PHBs are highly conserved proteins that belong to the Stomatin-Prohibitin Flotilin-HfiC/K (SPFH) Protein superfamily. Here, we present the first report of these proteins in the parasite *T*. *cruzi*. PHBs bind to the mitochondria membranes forming ring complexes that stabilize these organelles. PHB1/2 complexes are considered as a novel type of membrane-associated chaperone/holdase [16] that stabilize newly translated mitochondrial gene products, suggesting that they are negative regulators of m-AAA protease [17]. Deletion of either one of the prohibitins in yeast results in an increased turnover of unassembled Cox3 by m-AAA protease. Besides, the PHB1/PHB2 complex can also bind to Cox2p and Cox3p, facts that suggest a chaperone role rather than that of protease inhibitor [18].

Prohibitins have also been observed associated with the nuclear and cytoplasmic membranes, where they seem to participate in cell signaling [19–21]. The participation of PHBs in multiple essential biological processes suggests a critical pleiotropic role for these proteins [22–23].

Our microscopy studies in *T*. *cruzi* indicate that PHBs have a stage-specific localization. In the epimastigote forms, PHB1 and PHB2 are present in the mitochondria, the flagellar pocket and the apical pole, and eventually clustered in the vicinity of the nucleus. However, in the infective trypomastigote and amastigote stages, both proteins are located next to each other in the parasite´s plasma membrane. In the procyclic trypomastigote forms of *T*. *brucei*, PHB1 localizes in the mitochondrial membrane, playing a possible role in the stabilization of the newly synthesized proteins in this organelle [13]. However, in *L*. *donovani*, PHB1 localizes in the aflagellar pole and the flagellar pocket of the infectious promastigote forms, and a role in macrophage-parasite interactions has been assigned [12]. We previously observed that PHB1 of *L*. *major* PHB1 is present in the membrane and the vicinity of the mitochondria, whereas PHB2 is situated in the mitochondria and the nuclear membrane. The different locations and the multiple proposed functions[14], is in agreement with the need of *T*. *cruzi* to adapt to different environments throughout its life cycle agree with the pleiotropic role of PHBs.

In *T*. *cruzi*, the overexpression of PHB1 and PHB2 did not have an effect on the cell growth of epimastigotes (Fig 9D), but affected the process of metacyclogenesis. In cells overexpressing PHB1, we observed a higher differentiation percentage when compared to those overexpressing PHB2, and both cell lines achieved higher differentiation percentages than the control (Fig 5E). During metacyclogenesis, the parasites undergo a series of biochemical [24] and morphological changes [25] inside the insect gut, where the production of ROS is part of the insect’s defenses against parasites. The pro-oxidant molecules generated in the presence of the heme-groups (product of the hemoglobin digestion) stimulate the proliferation of the epimastigotes, whereas antioxidants such as N-acetyl cysteine (NAC) and urate induce metacyclogenesis *in vivo* [26]. Other factors such as sugar depletion, the increase in ionic strength, culturing in defined media enriched with some aa (TAU) [27,28] and an increase in cAMP levels favor the metacyclogenesis process [29]. Da Silva et al. (2015) showed that hemegroups can bind to the IF2α kinase catalytic center blocking its activity, causing the accumulation of H_2_O_2_ with the regulation of the superoxide dismutase. These changes inhibit the growth of the epimastigote forms, favoring metacyclogenesis [30].

*T*. *cruzi* has a very complex life cycle [31] and the wide variety of environments faced by the parasite should be reflected in the multiple adaptive physiological and biochemical changes to survive in these environments. The oxidative stress inside the vertebrate and invertebrate hosts is one of the major factors that the parasite must counteract through its antioxidant defenses [32]. In humans [33] and *L*. *major* [14], it was proposed that PHBs could act as intracellular ROS scavengers, protecting against this oxidative stress [34]. Several studies showed that *T*. *cruzi* amastigotes with increased activity of the enzyme 8-oxo-dGTPase multiply faster than wild-type parasites [35–37]. These facts indicate that PHBs, rather than participating in cellular infection, favor the intracellular multiplication of amastigotes by facilitating their survival in an environment with ROS (Fig 10 B and 10 C).

The ability of activated macrophages to destroy the parasites is associated with the generation of H_2_O_2_ [38]. In animal cells infected with *T*. *cruzi* overexpressing tryparedoxin peroxidase caused an increase in the infection rates [39]. Using cardiomyocyte cells in *in vitro* and *in vivo* models, Pereira et al. (2017) [40] described how histiotropism could be mediated by the ROS level produced by these cells, and how inhibition of the oxidative stress affects the rate of intracellular multiplication in some *T*. *cruzi* strains. *T*. *cruzi* strains overexpressing the TcMTH enzyme [41], responsible for removing the 8-oxo-dGTP produced by the oxidative damage, multiply more actively in the macrophage cytoplasm than the wild strain, a fact that agrees with our PHB overexpression experiments, and it is likely to be due to their increased capacity to eliminate the ROS generated by the cell.

The fact that PHB1 and PHB2 are close to each other on the surface of trypomastigotes of *T*. *cruzi* (Fig 7) suggests the possibility that they act directly against the ROS generated by the host cell.

The presence of phosphorylation sites next to the PHB2 iron-binding domain of *T*. *cruzi* suggests that the phosphorylation of this site may be related to cell signaling processes. In particular, the phosphorylation of tyrosine residues within this domain may affect its capacity to bind iron, thus regulating the intracellular traffic of this ion. The ability to bind and sequester iron ions by PHBs would prevent the formation of ROS generated by the free form of the ion and, consequently, avoid damage to the DNA (Fig 8). In agreement with these facts, our supercoiled plasmid DNA protection experiments demonstrated that *T*. *cruzi* PHBs exert a protection effect against ROS induced by iron [42–44].

To assess the importance of PHBs for *T*. *cruzi* we used the CRISPR/Cas9 technology to attempt to knock out both genes of this parasite. Although we could not detect changes in the PHB1 target DNA sequence or PHB1 gene locus (S8 Fig), we observed a reduction in PHB1 protein levels. This fact encouraged us to continue analyzing the expression rates of PHB1 of transfected cells. Similar phenomena were described by Lander et al. (2016) [45] when transfecting *T*. *cruzi* cells with the pTREX/cas9 vector containing the sgRNAs that targeted the *prf1* and *pfr2* genes (paraflagellar rod). Although they did not detect any mutation in the target sequence, they were able to analyze the effect of the lack of both proteins and the detachment of the flagellum from the body of the protozoon. These authors observed considerable chromosomal rearrangements in the transfected clones and suggested that the genome of the parasite was reorganized in such a way that copies of the gene were transferred to places where Cas9 could not reach them, and in these new locations, the genes would not be expressed or have a reduced expression. In our case, the PHB1 gene remained intact and we could not demonstrate mobilizations to other regions of the genome. The transcription and translation of the gene were reduced without apparently affecting other cell physiologic parameters like cell growth. Due to the lack of non-homologous end-joining recombination in *T*. *cruzi* and the presence of very rigorous homologous recombination the possibility of introducing indels is difficult, and it could only be possible by microhomology recombination [46]. We can not also rule out that changes due to pTREX plasmid insertion may have caused an alteration in the expression of PHB1 gene.

For PHB2, we replaced the gene with a blasticidin antibiotic marker but, since the mutated cells were short-lived, we could only study the phenotypic effects by TEM. Thus, in *T*. *cruzi*, the *phb2* gene is essential for the survival of the organism. A similar effect has been reported in *C*. *elegans*, where the depletion of the PHBs shortens the lifespan of this organism. Also, in *S*. *cerevisiae*, a disruption of the PHB complex decreases the replicative capacity of this microorganism [47–49] and induces morphological changes characteristic of aging. The complete depletion (yeast null mutants) leads to a mitochondrial alteration that causes a loss of respiratory capacity [50]. These observations agree with our results, i.e., the lack of PHB2 altered the mitochondrial morphology, induced vacuolation of the cytoplasm and caused the cell death of *T*. *cruzi*.

In summary, we report that *T*. *cruzi* PHB1 and PHB2 have a stage-specific location and, although the overexpression of PHBs did not increase the epimastigote´s multiplication capacity or the trypomastigote´s infection capacity, it had a clear effect in the parasitization index and differentiation into the metacyclic stage. We demonstrated that a reduction on PHB1 expression increased the parasite’s susceptibility to ROS and that the absence of PHB2 has a lethal effect on the parasite. These two results suggest that PHBs could act as ROS protectors. In conclusion, PHBs have a role in metacyclic differentiation, amastigote replication and ROS detoxification in the parasite *T*. *cruzi*.

## Materials and methods

### Ethics statement

Experiments using animals were performed following the institutional guidelines (Spanish government regulations (Real Decreto RD1201/05)) and the guidelines of the European Union (European Directive 2010/63/EU). These experiments were approved by the Ethical Committee of the University of Granada (235-CEEA-OH-2018) and by the authorities of the Regional Government of Andalucía (JJAA) (number 12/11/ 2017/162).

### Vero cell culture

Vero cells (ECACC 84113001) were cultured in MEM medium supplemented with 10% heat-inactivated (30 min at 56°C) fetal bovine serum (iFBS) (Gibco) and incubated at 37°C in a moist atmosphere enriched with 5% CO_2_. Nunc flasks of 25 cm^3^ or 75 cm^3^ were used for the cell culture, as previously described [51].

### Parasite strains and culture

Epimastigote stages of *T*. *cruzi* from the strains: DM28c (DTU I), Pan 4 (DTU I), Y (DTU II), 3663 clones (DTU III), 4167 clone (DTU IV), MNCl2 (DTU V) and CL Brener (DTU VI) were maintained by weekly passages in liver infusion tryptose (LIT) culture medium [52] supplemented with 10% iFBS (Gibco, US) at 28°C. Cultures of the epimastigotes in logarithmic growth phase were used in all of the experiments.

Metacyclic trypomastigote forms were obtained by inducing metacyclogenesis in the epimastigote stages. Briefly, epimastigotes were collected by centrifugation at 1,500 *x g* for 10 min and washed two times in PBS. Then, they were resuspended (5x10^8^ cells/mL) in triatomine artificial urine (TAU) culture medium [53] and incubated for 2 h at 28°C. After the incubation, they were diluted to 5x10^6^ parasites/mL in TAU3AAG medium (TAU supplemented with 10 mM L-proline, 50 mM L-sodium glutamate, 2 mM L-sodium aspartate, and 10 mM D-glucose) and cultured for 72 h at 28°C [53,54]. Once the transformation from epimastigotes to the metacyclic forms took place, the parasites were collected to infect synchronized Vero cell cultures following the methodology described by Osuna et al. [55]. After 100 h post-infection, the trypomastigote forms were released from Vero cells and collected by centrifugation using the same conditions previously described. For the purification of *T*. *cruzi* intracellular amastigotes, we followed the protocol described by Marques et al (2011) [56].

### Growth curves

The different strains and clones of *T*. *cruzi* were cultured in LIT medium for 8 days at 28°C using an initial density of 1x10^6^ parasites/mL. Growth was measured daily by counting the number of parasites using a Neubauer hemocytometer chamber. Counts were performed in triplicate.

### Cloning and expression of recombinant PHBs in bacteria

*T*. *cruzi phb*1 (TCDM_03905) and *phb*2 (TCDM_03718) genes were amplified using the primers 1 and 2, and 3 and 4, respectively (S1 Table). As template, the DNAs from the latter reactions were employed. The PCRs were performed following these conditions: an initial incubation for 5 min at 95°C, followed by 40 cycles of denaturing at 95°C for 1 min, annealing at 62–64°C for 1 min and extension at 72°C for 1 min, and a final extension of 10 min at 72°C. The amplification products were cloned using the pGEM-T Easy vector (Promega, Madrid, Spain) and then sub-cloned in the pQE30-Xa expression vector (Qiagen, Hilden, Germany), using the restriction enzymes *Bam*HI-HF and *Sal*I-HF (New England Biolabs, Ipswich, MA USA).

*Escherichia coli* strain M15 (Qiagen, Hilden, Germany) was transformed with the recombinant plasmids following the manufacturer’s instructions. Overexpression was induced by the addition of isopropyl β-D-1-thiogalactopyranoside (IPTG) 1 mM (Sigma, Madrid, Spain) for 4 h. The production of the recombinant proteins PHB1 [30.9 kDa PHB1 + 6 kDa histidine tag) and PHB2 (33.2 kDa PHB2 + 6 kDa histidine tag) was confirmed by Western blot using an antibody against the histidine tag as described below (Sigma, Merck Life Science S.L.U. Spain). Once the culture was centrifuged, the bacterial pellet was resuspended in bacterial lysis buffer (7.5 mL of buffer per gram of centrifuged bacteria), to which 0.35 mg / mL of lysozyme were added, and incubated for 30 min at 20°C. Subsequently, 10 μg / mL RNase, 5 μg / mL DNase and 6 mM MgCl_2_ and protease inhibitors and Triton X-100 were added to a final concentration of 5%. and incubated for 20 min. The samples were sonicated in a Branson SLP Sonifier (intervals of 10 s for a total of 2 min, with a 10 s pause) and then centrifuged at 12,000 *x g* for 20 min at 4°C and the pellets were resuspended in lysis buffer (50mM Tris-HCl pH 8.0, 500mM NaCl, 10mM EDTA, 5mM β-mercaptoethanol) with a final concentration of 2% Triton X-100. The sonication and centrifugation steps were repeated, also adding lysis solution with final concentrations of 1 and 0.5% Triton X-100. After performing 3 washes with PBS, the pellets were stored at -20°C until use.

Since the chromatographic purification of recombinant prohibitins using nickel columns was not satisfactory, the proteins were purified by elution from polyacrylamide gels following Thermo Scientific protocols [57]. This was performed to obtain a single band of the recombinant proteins to be employed for the subsequent immunization protocol. The eluted proteins were then concentrated using Amicon columns of 10 kDa (Merck Millipore, Merck KGaA, Darmstad, Germany).

### Polyclonal antibody production

Two four-week old male Wistar rats were immunized with 20 μg of each of the recombinant proteins, PHB1 and PHB2. Besides, two five-week old male BALB/c mice were immunized with 10 μg of the recombinant PHB2. Each antigen was administered intraperitoneally, using a suspension of the antigen diluted in PBS plus Freund’s adjuvant (Sigma, Madrid, Spain) in a 1:1 ratio. Freund’s complete adjuvant was employed for the first inoculation and was then switched to incomplete Freund’s adjuvant for the subsequent immunizations (5 weeks). The animals were exsanguinated two weeks after the final injection and the reactivity of the final antibodies was evaluated by ELISA and Western blot assays. As negative controls, pre-immune sera obtained from the same animals before the immunization process were employed. Specificity controls of the antibodies obtained against the recombinant proteins were performed by Western blot, comparing the sensibility and specificity of the immune sera as described below.

### Western blot analysis for the detection of PHB1 and PHB2 in trypomastigotes, epimastigotes and amastigotes of *T*. *cruzi*

For the Western blots, 30–40 μg of protein samples from lysates of each stage of the parasite, quantified by the Bradford method (Sigma, Merck Life Science S.L.U, Spain), were electrophoresed on 12% SDS-PAGE gels and transferred to PVDF membranes (Bio-Rad, Alcobendas, Madrid) using a Trans-Blot Turbo Transfer System (Bio-Rad, Alcobendas, Madrid). The Precision Plus Protein All Blue (Bio-Rad, Alcobendas, Madrid) or the Page Ruler Prestained Protein Ladder (Thermo, Alcobendas, Spain) were the molecular weight markers included. After the protein transfer, membranes were immersed in blocking buffer (PBS, 0.1% Tween 20 and 4% non-fat dry milk) and incubated overnight under orbital agitation at 4°C. The primary antibody was added at the appropriate dilution in blocking buffer over the membrane and incubated for 2 h at room temperature, following a protocol described elsewhere [18]. PHBs were recognized by the antisera obtained in rats (anti-PHB1 and anti-PHB2) as described below, using dilutions of 1/6,500 and 1/17,000, respectively. The histidine tag of the recombinant proteins was detected using the poly-histidine tag (6-His) antibody (MyBioSource, San Diego, CA. USA) (1/1.000 dilution) and the TAPtag was identified using the CBP Tag mAb mouse antibody (GenScript, Piscataway, NJ, USA) (1/1,000 dilution). As loading controls, the detection of GAPDH was performed using an anti-GAPDH antibody produced in rabbit (Sigma, Madrid, Spain) (1/5.000) and tubulin was detected with an anti-tubulin sheep polyclonal antibody (Cytoskeleton, Denver, CO. USA) (1/1,000). After incubation with the primary antibodies, the membranes were washed four times in washing buffer for 10 min and then incubated with the secondary antibodies labeled with HRP for 1 h at room temperature. After several washes, the reaction was visualized using Clarity Western ECL Substrate (Bio-Rad, Alcobendas, Madrid). The results of the immunoblots were visualized in a ChemiDoc MP Imaging System (Bio-Rad, Feldkirchen, Germany). Image acquisition was carried out by the device, through Electrophoresis Multiple channel methods where channel 1 was a colorimetric with white light illumination by means of Epi illumination and mode 2 was Automatic Chemo Exposure, and both images were superimposed by the device. The marker proteins were colored markers page Ruler (Thermo Fisher).

### cDNA synthesis and real-time PCR (RT-qPCR)

The mRNA expression for PHB1 and PHB2 was quantified in different stages of *T*. *cruzi* (trypomastigote, epimastigote and amastigote) by quantitative RT-PCR (qRT-PCR). Total mRNA was isolated from the *T*. *cruzi* forms using the RNeasy and the Oligotex mRNA kit (Qiagen, Hilden, Germany). cDNA synthesis was carried out using the QuantiTect Reverse Transcription Kit (Qiagen, Hilden, Germany), following the manufacturer´s protocol. RT-qPCR was employed to determine the expression levels of native PHB1 and PHB2 proteins of epimastigotes and trypomastigotes using the ΔΔCT method and GAPDH as the reference gene. Quantitative PCR analysis (RT-qPCR) was performed as described previously [58]. The pairs of primers employed for the RT-qPCRs (primers 5–8) are listed in S1 Table.

### Confocal laser microscopy

In order to localize the mitochondria in the different stages of the parasite, the samples were incubated with MitoTracker red CMXRos (Invitrogen, Thermo, Alcobendas, Spain) following the manufacturer’s instructions. Then, the parasites were rinsed three times in PBS for 5 min and fixed with 3.7% paraformaldehyde for 15 min at 4°C, for their subsequent permeabilization with PBS-0.1% Triton X-100 for 5 min and the blocking step in PBS-1% BSA (Sigma, Madrid, Spain) for 1 h at room temperature. PHBs were localized using the anti-PHB1 (1:100 dilution, produced in rat) and the anti-PHB2 (1:200 dilution, produced in mouse) antibodies, followed by incubation with FITC anti-rat IgG antibodies (whole molecule; 1:350 dilution) (Sigma, Madrid, Spain) or Alexa Fluor 647 goat anti-mouse IgG antibodies (H+L, 1:350 dilution) (Life Technologies, Carlsbad, CA, USA). For nuclear and kinetoplast DNA staining, fixed samples were incubated for 15 min in a 10 μg / mL DAPI solution. The stained samples were preserved using the Ibidi mounting medium (Ibidi, Gräfelfing, Germany) and examined under a Leica DMI6000 confocal laser microscope equipped with a filter system for FITC (mean wavelength 530 nm, maximum 490 nm).

To evaluate the presence and localization of the PHBs in the intracellular amastigote stage, Vero cells were cultured in chambered coverslips (Ibidi) and then infected in a 5:1 ratio of trypomastigotes: mammalian cells. After two hours of infection, the extracellular parasites were removed by washing the cell cultures with fresh culture medium and then incubated for 72 h under the conditions previously described. Subsequently, at 12 h of incubation, the coverslips with cells were washed in PBS, fixed and prepared for microscopy as previously described, in order to study the infection levels of the parasites transfected with the *phb1* and *phb2* gene and the empty plasmid (pTREX) and compare to the infection and multiplication rates obtained by the wild-type non-transfected strain. Observation fields were randomly selected for each coverslip and 350 cells were counted in each preparation.

After following the progress of the cultures by optic microscopy and prior to the transformation of amastigotes into trypomastigotes (72 h post infection), the preparation for confocal microscopy was repeated to determine the location of fluorescence of the PHBs in the amastigotes and to calculate the percentage of parasitized cells and the number of amastigotes per cell. The experiments were performed in triplicate.

### Transmission electron microscopy

Epimastigotes and trypomastigotes of *T*. *cruzi* Dm28c-WT were fixed overnight, at 4°C, using 2% glutaraldehyde and 2% formaldehyde in 0.1 M cacodylate buffer (pH 7.2). Then, the biological material was embedded in Epon resin. Ultrathin sections were stained with 8% uranyl acetate, followed by 0.2% lead citrate and examined under a LIBRA 120 PLUS Carl Zeiss SMT microscope (Carl Zeiss, Oberkochen, Germany).

### Immuno-electron microscopy

Approximately 1x10^9^ epimastigotes or trypomastigotes were centrifuged and washed twice in 0.2 M phosphate buffer (pH 7.2) (PB) and then fixed in 0.5% glutaraldehyde, 2% paraformaldehyde in PB buffer (pH 7.2) for 2 h at 4°C. The samples were washed three times in PB, embedded in LR-White resin and sliced into 100-nm-thin sections using an ultramicrotome (Leica, Wetzlar, Germany). For the immunogold labeling, the samples were incubated with the anti-PHB1 (produced in rat) (1:100) and/or the anti-PHB2 (produced in rat or mice) (1:150; 1:100) antibodies, followed by incubation with the 10 nm and/or 18 nm gold-conjugated secondary antibodies (Sigma, Madrid, Spain), respectively. Ultra-thin sections were observed under a LIBRA 120 PLUS Carl Zeiss SMT microscope (Carl Zeiss, Oberkochen, Germany). As control of the specificity of the antibodies and to determine the location of the overexpressed proteins, once the epimastigotes of the transfected parasites were fixed, embedded in LR-White resin and sliced, they were treated with protein A labelled gold particles instead of performing the incubation with the specific antibodies. These particles were employed since the Fc domain present in the vector has affinity to protein A.

## Immobilized metal ion affinity chromatography (IMAC)

For these assays, a culture of epimastigote forms (7.5x10^6^/mL) at the end of the exponential growth phase was employed. The parasites were harvested by centrifugation and the pellets were washed three times in PBS, resuspended and lysed in a phosphate buffer (pH 7.4) containing 0.25 mM sucrose, 1 mM EDTA, 1 mM, DTT, 1% Triton X-100 and a protease inhibitor cocktail (Complete Mini, Boehringer Mannheim GmbH, Mannheim, Germany). The samples were finally frozen and thawed three times, followed by sonication in a Branson SLP Sonifier (intervals of 10 s for a total of 2 min, with a 10 s pause).

Modified prepacked HiTrap columns (GE Healthcare, Pittsburgh, PA, USA) linked to Ni Sepharose 6IMAC were used to obtain Fe^3+^ Sepharose-immobilized columns by substituting the Ni^2+^ for Fe^3+^ ions, according to the methodology described [59]. Briefly, after washing the original Ni (II) Sepharose column with three volumes of acetonitrile, the column was washed with three volumes of a 100 mM EDTA solution in water with a regular flux of 10 μL / min for 10 min to remove the Ni (II) linked to the Sepharose. After several washes in deionized water, the column was activated with an acid buffer (0.02M sodium acetate, 0.5M NaCl, pH 4) and subjected to further washes with water to remove excess of acetate in the exclusion volume. A 100 mM FeCl_3_ solution (pH 3) was passed through the column, maintaining a flow of 10 μL / min for 10 minutes. The column was washed again with distilled H_2_O to remove excess of the metal ions and subsequently, the acid buffer was passed until the pH of the effluent was equal to the pH of the wash buffer. This buffer allows to elute Fe ions that are not strongly bound so that they do not bind during the adsorption / desorption of the proteins. The column was equilibrated with binding buffer (0.02 M sodium phosphate; 0.8 M NaCl pH 6.8) [60].

The extracts of the total proteins obtained were diluted in binding buffer containing 1% Triton X100 plus a protease inhibitor cocktail (Complete Mini, Boehringer Mannheim GmbH, Mannheim, Germany) and kept in recirculation through the column for 6 h. Then, the column was washed with binding buffer, and the retained proteins were eluted with a buffer containing 0.05M EDTA, 0.5M NaCL pH 7.

After each run, the IMAC columns were stripped off the immobilized Fe^3+^ ions by leaving the medium in 0.05 M EDTA overnight, and then recharged with new Fe^3+^ ions. To detect the PHBs after the elution process, proteins bound to Fe^3+^ were precipitated with acetone, electrophoresed in an SDS-PAGE gel and transferred to a nitrocellulose membrane for Western blot analysis. The membranes were incubated with the anti-PHB1 and anti-PHB2 antibodies and then with the secondary antibodies, as previously described.

### In silico analysis

The TritrypDB and NCBI (https://www.ncbi.nlm.nih.gov/) databases were used to retrieve the PHB sequences. CRISPR guide RNAs were designed using EuPaGDT [61]. InterPro (https://www.ebi.ac.uk/interpro/) was employed for the protein classification; IonCom (https://zhanglab.ccmb.med.umich.edu/IonCom/) for predicting small ligands, including the metal and acid radical ion binding site, and Galaxy 39 (https://usegalaxy.org/) for the prediction of the Fe^+3^ binding motifs. Phobius, (http://phobius.sbc.su.se/), TOPO2 (http://www.sacs.ucsf.edu/TOPO2/) and CCTOP (http://cctop.enzim.ttk.mta.hu/) were employed to predict the protein topology; CSS-Palm (http://csspalm.biocuckoo.org/) for the protein palmitoylation site prediction; Sulfinator (https://web.expasy.org/sulfinator/) for the tyrosine sulfating sites prediction and myhits (https://myhits.isb-sib.ch/) for the prediction of the protein sequences and motifs in general. Clustal Omega (https://www.ebi.ac.uk/Tools/msa/clustalo/) and GeneDoc software were used for sequence alignments.

*T*. *cruzi* (TCDM_03905), *T*. *brucei* (Tb927.8.4810), *L*. *major* (LmjF.16.1610), *L*. *donovani* (LdBPK_161710.1) and *H*. *sapiens* (GI: 528281407, NP_001268425) were the IDs for sequence comparisons of PHB1 proteins. *T*. *cruzi* (TCDM_03718), *T*. *brucei* (Tb927.10.4310), *L*. *major* (LmjF.35.0070), *L*. *donovani* (LdBPK_350070.1) and *H*. *sapiens* (GI: 119609105, EAW88699) were the IDs for sequence comparisons of PHB2 proteins.

### Expression of recombinant PHBs in *T*. *cruzi*

The *phb1* (primers 9 and 10) and *phb2* (primers 11 and 12) (S1 Table) amplicons were inserted into the vector pENTR3C using the BamHI-HF/XhoI restriction sites. The genes were transferred to the integrative pTREX-TAPtag-GW vector [62], derived from the plasmid pTREX [63] and are able to integrate into the *T*. *cruzi* genome due to the presence of the ribosomal promoter. It also includes the HXI and GAPDH regions, which increase the expression of the protein of interest. Likewise, the plasmid with the GATEWAY cassette, which contains a recombination site that facilitates the insertion of the gene of interest and a tag called TAPtag (~21kDa) [62] was employed, using Gateway LR Clonase II enzyme mix (Invitrogen, Thermo, Alcobendas, Spain) following the manufacturer’s instructions and the Gateway cloning protocol (Life Technologies-Invitrogen, Thermo, Alcobendas, Spain). The resulting plasmids pTAPHB1 (containing phb1), pTAPHB2 (containing phb2) and pTAP (empty pTREX-TAPtag-GW as a control) were transfected into the *T*. *cruzi* Dm28c strain by electroporation, according to a previously described protocol [63]. Transfected parasites were selected after ~6 weeks of culture in the presence of 200 μg/mL of Geneticin (G418) (Sigma, Madrid, Spain).

### CRISPR/Cas9 gene editing

To carry out the disruption of the *phb*1 and *phb*2 genes, the CRISPR/Cas9 methodology was employed. The sgRNAs were designed using the bioinformatic Eukaryotic Pathogen CRISPR guide RNA/DNA Design Tool. [61]. For each gene, two different sgRNAs were selected, each one containing a 19–22 nt protospacer complementary to the target gene, followed by the Protospacer Adjacent Motif sequence (PAM), NGG required for the recognition of the Cas9 enzyme from *S*. *pyogenes*.

The sgRNAs were amplified using as template the pUCsgRNA plasmid, which also contains the sgRNA backbone sequence (82 bp). For the PCRs, the forward primers employed were 13 and 14 for *phb*1, and 15 and 16 for *phb*2 (S1 Table). The common reverse primer was primer 17, and the reaction was carried out following a previously described protocol [64]. All of the designed primers contained a *Bam*HI restriction site for cloning into the Cas9/pTREX-n vector. As control, a scrambled sgRNA (scCas9) was employed. In this case, the protospacer was replaced with an irrelevant sequence of 20 scrambled nucleotides that did not target any gene in the *T*. *cruzi* genome [64].

For the gene replacement experiments, a donor DNA containing the blasticidin resistance gene flanked by ~500 bp of the 5´and 3´ UTR regions from the *phb2* gene was constructed by PCR. For the amplification of the *phb*2 5`UTR segment, primers 18 and 19 were employed, whereas the 3`UTR segment was amplified with primers 20 and 21 (S1 Table). For the amplification template, gDNA from *T*. *cruzi* strain DM28c was employed. The blasticidine resistance gene was amplified from pTREX-b with primers 22 and 23.

For checking the integrity of the PHB1 gene locus after the transfection with 1sgCas9PHB1 and 2sgCas9PHB1, two pairs of primers were designed for amplifying upstream (numbered as 24–25) and downstream (numbered as 26–27) of the ORF of the gene.

Epimastigote forms of *T*. *cruzi* DM28c at the exponential growth phase were transfected with the plasmids containing the different sgRNAs. The co-transfection with the blasticidine linear donor DNA was performed using a one-step transfection protocol as previously described [33]. After 24 h post-transfection, the selection antibiotics (200 μg/mL of G418 and/or 10 μg/mL of blasticidine) were included in the medium and selection was allowed for approximately 2 weeks. The transfection was performed in triplicate.

### Transfection and cloning of the epimastigote stages

A culture of epimastigotes at the logarithmic phase of growth was employed. Once washed in PBS, they were counted, adjusted to 3.10^7^ parasites / mL in 350 μL of hypoosmolar buffer (Eppendorf) and placed into cuvettes of 2 mm electroporation (Eppendorf) to which 20 μg of the plasmid DNA were added (pTREX with the different constructs described above and empty pTREX as a control). Transfections were performed in an Eppendorf multiporator 4308 device, under the following conditions: 100 μs, 500 V, and 2p (pulses). Once electroporated, the parasites were placed into 25 cm^2^ Roux flasks (Nunc) with 5 mL of LIT culture medium supplemented with 10% IFCS. After 24 h at 28° C for culture stabilization, the selection antibiotic G418 (Sigma) was added at a concentration of 200 μg / mL. The medium with the antibiotic was renewed weekly until stable growth populations were achieved, after approximately 6 weeks.

Once the stable populations of the transfected epimastigote lines were obtained, the cloning process was carried out, following the limit dilution procedure according to the technique previously published [58] and adjusting the number of parasites to 1 epimastigote / 10 μL. The parasites were then placed in the base of each well of a flat-bottom 96-well (Nunc) microtiter plate and microscopically corroborated at 2 h after placing the suspension medium in the wells of the plate. Wells with more than one epimastigote were discarded. 50 μL of modified LIT medium were added to the wells. These modified LIT medium consisted of 50% sterile culture medium (the same culture medium that previously supported the growth of the parasites for at least 96 h, filtered through 0.22 μm pore filters) and 50% fresh medium. The plates were sealed and incubated at 28°C, with periodical addition of culture medium to generate a population of epimastigotes from a single cell (approximately 12 weeks).

### Quantification of reactive oxygen species (ROS) by flow-cytometry

After testing different H_2_O_2_ (0–800 μM) and parasite concentrations (1x10^5^-1x10^7^ cells/mL), the experiments for testing the ROS production were performed using 1x10^7^ epimastigotes/mL, 500 μM of H_2_O_2_ and 90 min as the incubation time. To measure the oxidative stress, the fluorescent probe 2’,7’-dichlorofluorescin diacetate (DCFDA) (Abcam, Cambridge, UK) was employed. Epimastigotes of *T*. *cruzi* (pTAPHB1), *T*. *cruzi* (pTAPHB2) and *T*. *cruzi* (pTAP) lines were labeled with the dye and incubated with 500 μM H_2_O_2_ for 90 min, as described by Lazarin-Bidóia et al. (2015) [65]. Parasite populations were analyzed in a FAC Scalibur Becton Dickinson flow cytometer (Becton Dickinson, Becton Drive Franklin Lakes, NJ USA). The mean fluorescence intensity (MIF) was determined in all cell lines (treated and non-treated with H_2_O_2_).

To evaluate the ability of PHBs to chelate Fe^3+^ ions and indirectly inhibit the Fenton reaction, (thus protecting DNA against oxidative stress), supercoiled forms of the plasmid pUC19 were employed, following a previously described protocol [66]. The reaction mixture consisted of pUC19 (10 nM), 50 μM FeCl_3_, 10 mM H_2_O_2_ and each of the PHB1, PHB2 or BSA (Sigma, Madrid, Spain) as protein control, at concentrations of 500, 250 and 125 μg/mL, respectively (final volume: 50 μL). Circular supercoiled plasmid pUC19 DNA was incubated with 50 μM FeCl_3_ and 10 mM H_2_O_2_ in the presence of variable concentrations of the recombinant proteins PHB1 and PHB2.

The supercoiled DNA was dissolved in a 10 mM Tris, 100 mM KCl solution (pH 7.4), to which the different concentrations of PHBs or BSA were added. After incubating the mixture at 20°C for 15 min, a solution of FeCl_3_ was added and the mixture was further incubated under the same conditions. Finally, 10 mM H_2_O_2_ were added and the mixture was incubated for 1 hour at 37°C. Samples were analyzed in 1.5% agarose gels at 100 V for 40 min to observe the effects of Fe^3 +^ plus H_2_O_2_ over the DNA.

### Statistical analyses

Experiments were performed in triplicate. Analysis or comparisons between groups were made using unpaired one-way ANOVA and *at posteriori* Fisher´s LSD or Tukey’s tests, using the GraphPad Prism 7 software (GraphPad Software, San Diego, CA, USA). Values of *p* < 0.05 were considered statistically significant.

## Supporting information

S1 Table Sequences of the oligonucleotides employed in this study. In underlined italics: restriction sites; in underlined bold: gene-specific homologous region (Protospacer).
(DOCX)

S1 Fig Multiple analysis by Clustal algorithm of PHB1 (A) and PHB2 (B) of T. cruzi, T. brucei, L. major, L. donovani and H. sapiens. The red line indicates the Band 7/ SPFH domain superfamily (IPR036013).
(TIF)

S2 Fig Specificity of the sera obtained against *T*. *cruzi´*s recombinant PHBs. (**A**) M: PageRuler Prestained Protein Ladder (Thermo); 1: total proteins of epimastigotes of *T*. *cruzi*; 2: total proteins of *L*. *major* promastigotes, 3: total proteins of the procyclic form of *T*. *brucei*; 4: Vero cell total proteins. (**B**) M: PageRuler prestained protein ladder (Thermo); 1: total proteins of *T*. *cruzi* epimastigotes; 2: total proteins of *L*. *major* promastigotes; 3: Vero cell total proteins; 4: total proteins of the procyclic form of *T*. *brucei*.
(TIF)

S3 Fig Relative expression of PHB1 (A) and PHB2 (B) versus tubulin in the epimastigote forms of T. cruzi classified in different DTUs. M: PageRuler prestained protein ladder (Thermo); total proteins of: 1: PAN4 strain (DTU I); 2: DM28c strain (DTU I); 3: Y strain (DTU II); 4: clone A3663 strain (DTU III), 5: clone 4167 strain (DTU IV); 6: MNCl2 strain (DTU V); 7: CL Brener strain (DTU VI). The ANOVA test did not reveal significant differences between the expression results of the parasites of different DTUs (p <0.05, ns: non-significant).
(TIF)

S4 Fig Negative controls of the immune-electron microscopy experiments. Control 1: parasites incubated with the preimmune serum and control 2: parasites incubated with serum from non-immunized animals.
(TIF)

S5 Fig Detection of PHB1 (A) and PHB2 (B) after the affinity chromatography on Sepharose charged with Fe^3+^ ions by Western blot. M: PageRuler prestained protein ladder (Thermo); 1: total proteins in the epimastigote lysate of the DM28c strain; 2: total epimastigote proteins after the passage through the affinity column; 3: wash 1; 4: wash 2; 5: wash 3; 6: product of the first elution; 7: product of the second elution; 8: product of the third elution.
(TIF)

S6 Fig Overexpression of the recombinant PHB1 (A) and PHB2 (B) in *T*. *cruzi* epimastigote (1), trypomastigote (2) and amastigote forms (3), using an anti-CBP (Abcam) as primary antibody that recognizes the TAPtag.
(TIF)

S7 Fig A. Circular map of the pTREX-TAPtag-GW vector: The GATEWAY (GW) cassette is shown in green and the TAPtag is shown in light blue, which consists of CBP (calmodulin-binding peptide), PA (protein A) and the cleavage site for TEV protease. The ribosomal promoter is seen in lilac and the HX1 region downstream in red. This plasmid possesses the resistance genes amp (ampicillin) and neo (neomycin). Flanking the neo gene are intergenic *gapdh* regions, according with [62,53]. B. Localization of PHB1 overexpressed in the epimastigote form and recognized by Protein A labeled with Au 20 nm. C. Localization of PHB2 overexpressed in the epimastigote form and recognized by Protein A labeled with Au 20 nm.
(TIF)

S8 Fig A. Schematic representation of the PCR strategy used to amplify the upstream and downstream regions of the PHB1 ORF. B. 1.5% Agarose gel showing the expected PCR products upstream (1) and downstream (2) the PHB1 locus in the PHB1 Clone 1 (CRISPR-Cas9 gene edited) and WT cell lines.
(TIF)
