# Supplementary material for: Characterization and functional analysis of the proteins Prohibitin 1 and 2 in Trypanosoma cruzi
Source: PLoS Negl Trop Dis. 2021 Apr 8;15(4):e0009322. doi: 10.1371/journal.pntd.0009322 (PMC8057595; doi:10.1371/journal.pntd.0009322)
Supplement: S1 Table — In underlined italics: restriction sites; in underlined bold: gene-specific homologous region (Protospacer). (DOCX) [file pntd.0009322.s001.docx]

**S1 Table 1. Oligonucleotides used in this study**.

| Nº | Primer | Sequence (5´ → 3´) |
| --- | --- | --- |
| 1 | **PHB1pQE30 F** | AAAAAA*GGATCC*ATGAGTTTGAAATTTCTTCGACATCTTATGACTGG |
| 2 | **PHB1pQE30 R** | AAAAAA*GGATCC*ATGAGTTTGAAATTTCTTCGACATCTTATGACTGG |
| 3 | **PHB2pQE30 F** | AAAAAA*GGATCC*ATGGCGAAGGCACCACCCGGACC |
| 4 | **PHB2pQE30 R** | AAAAAA*GTCGAC*TTACAGGCGCCTGCCATGAACG |
| 5 | **PHB1RealtimeF** | GGCAGTTGTGGCAGAGTACA |
| 6 | **PHB1RealtimeR** | CTTGCCAAACTGAATGTCCA |
| 7 | **PHB2RealtimeF** | ATCACCCAGATGAGCTTTGG |
| 8 | **PHB2RealtimeR** | TCACCTTCCGCAAGAAGAAT |
| 9 | **PHB1TAPtagF** | AAAAAA*GGATCC*ATGAGTTTGAAATTTCTTCGACATC |
| 10 | **PHB1TAPtagR** | AAAAAAC*TCGAGC*CGCCACTCAAGTTCTTCATGTTAAGC |
| 11 | **PHB2TAPtagF** | AAAAAA*GGATCC*ATGGCGAAGGCACCACCCGGACC |
| 12 | **PHB2TAPtagR** | AAAAAA*CTCGAG*CCCAGGCGCCTGCCATGAACGTCTGC |
| 13 | **1sgPHB1** | GTTTTCCCAGTCACGACGGATCC**GAAAAGCGGGCGGCTATTGTG** GTTTTAGAGCTAGAAATAGCAAGTTAAAATAAGGCTAGTCCGTTATCA  ACTTGAAAAAGTGGCACCGAGTCGGTGCTTTTTTGGATCCAAAAA |
| 14 | **2sgPHB1** | GTTTTCCCAGTCACGACGGATCC**GGTGGACATTCAGTTTGGCA** GTTTTAGAGCTAGAAATAGCAAGTTAAAATAAGGCTAGTCCGTTATC  AACTTGAAAAAGTGGCACCGAGTCGGTGCTTTTTT GGATCCAAAAA |
| 15 | **1sgPHB2** | GTTTTCCCAGTCACGACGGATCC**GGCGCAACAGATGGCAGAGC** GTTTTAGAGCTAGAAATAGCAAGTTAAAATAAGGCTAGTCCGTTATCAA  CTTGAAAAAGTGGCACCGAGTCGGTGCTTTTTTGGATCCAAAAA |
| 16 | **2sgPHB2** | GTTTTCCCAGTCACGACGGATCC**GTCTTCTTGCGGAAGGTGAGG**  GTTTTAGAGCTAGAAATAGCAAGTTAAAATAAGGCTAGTCCGTTATCAA  CTTGAAAAAGTGGCACCGAGTCGGTGCTTTTTT GGATCCAAAAA |
| 17 | **pUCcrisperR** | CACCGAGTCGGTGCTTTTTTGGATCCACTG |
| 18 | **PHB2Bsd5`UTR F** | TTGACCTGGGGTTCTCTGTACTTC |
| 19 | **PHB2BsdEcoRI5`R** | AAAAAAGAATTCGTGCTCTTGCCGCTAAGACAAGAG |
| 20 | **PHB2Bsd3`BamHI F** | AAAAAAGGATCCGAAGGAGTGGAATGGAATGAAAAG |
| 21 | **PHB2Bsd3`UTR R** | TCAAAGTGCAGGGTACCCAAGACG |
| 22 | **BsdEcoRI F** | AAAAAAGAATTCATGGCCAAGCCTTTGTCTCAAG |
| 23 | **BsdBamHI R** | AAAAAAGGATCCTTAGCCCTCCCACACATAACC |
| 24 | **278.PHB1 IGR-ORF F** | CAAACAATAAACAGCACGACA |
| 25 | **279.PHB1 IGR-ORF R** | CAGTGAAAGATCCTCCAGGAC |
| 26 | **280.PHB1 ORF-IGR F** | GGAATGGACTACGATGAAC |
| 27 | **281.PHB1 ORF-IGR R** | ACGGCTCTGTTGTTAGTTG |
