# Supplementary figures and images for: Characterization and functional analysis of the proteins Prohibitin 1 and 2 in Trypanosoma cruzi
Source: PLoS Negl Trop Dis. 2021 Apr 8;15(4):e0009322. doi: 10.1371/journal.pntd.0009322 (PMC8057595; doi:10.1371/journal.pntd.0009322)

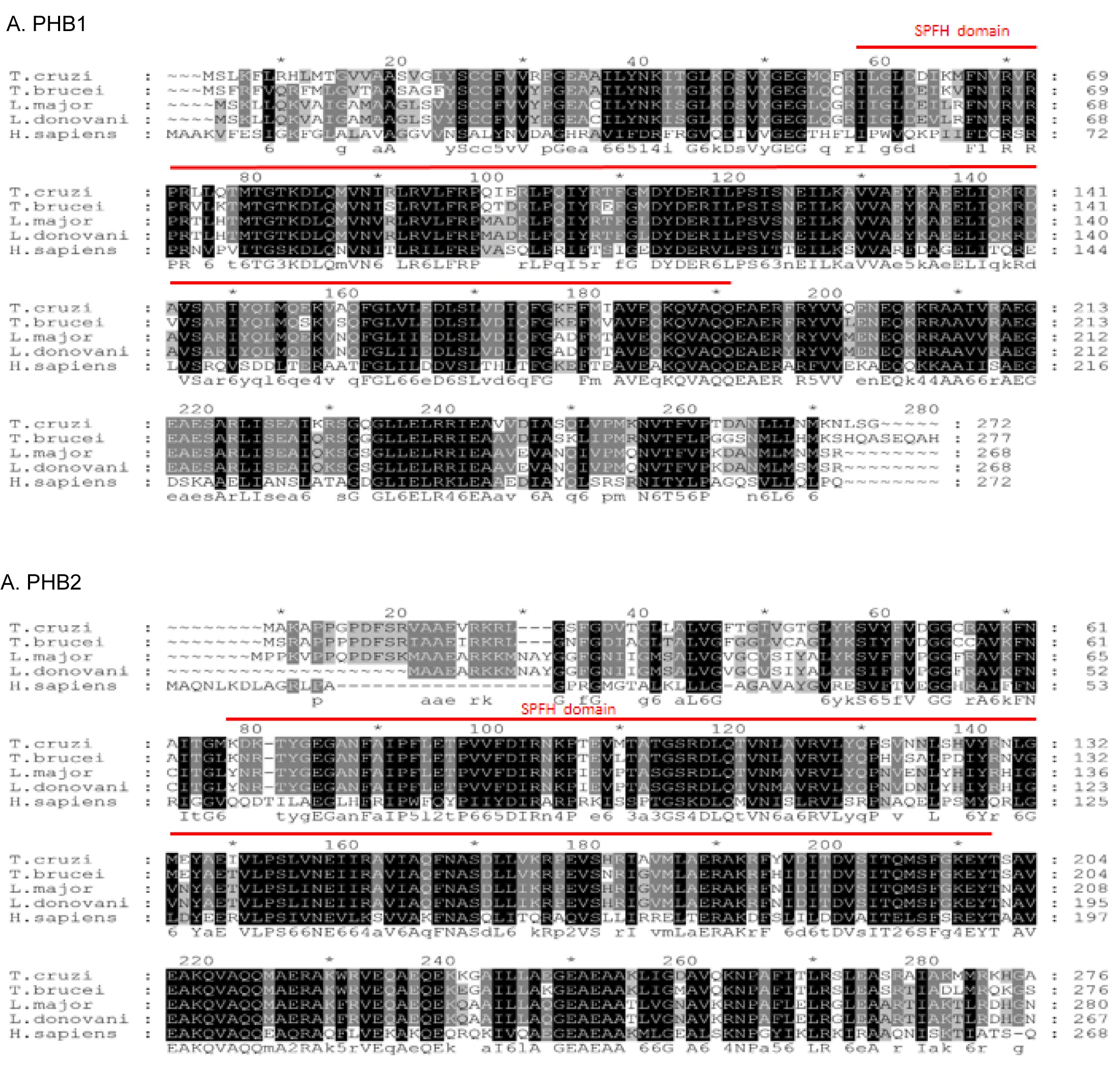

Supplement: S1 Fig — Multiple analysis by Clustal algorithm of PHB1 (A) and PHB2 (B) of T. cruzi, T. brucei, L. major, L. donovani and H. sapiens. The red line indicates the Band 7/ SPFH domain superfamily (IPR036013). (TIF) [file pntd.0009322.s002.tif]

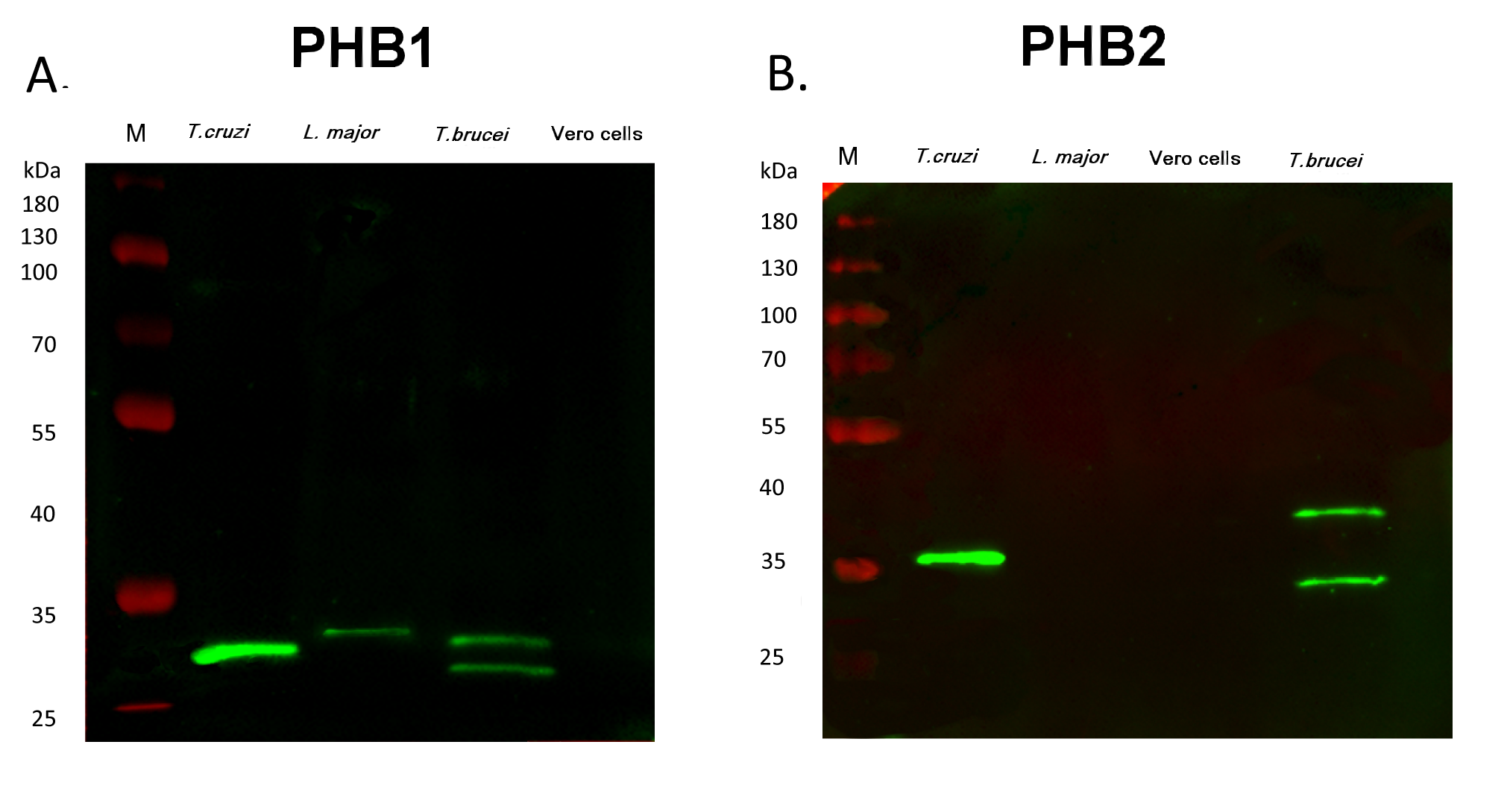

Supplement: S2 Fig — (A) M: PageRuler Prestained Protein Ladder (Thermo); 1: total proteins of epimastigotes of T. cruzi; 2: total proteins of L. major promastigotes, 3: total proteins of the procyclic form of T. brucei; 4: Vero cell total proteins. (B) M: PageRuler prestained protein ladder (Thermo); 1: total proteins of T. cruzi epimastigotes; 2: total proteins of L. major promastigotes; 3: Vero cell total proteins; 4: total proteins of the procyclic form of T. brucei. (TIF) [file pntd.0009322.s003.tif]

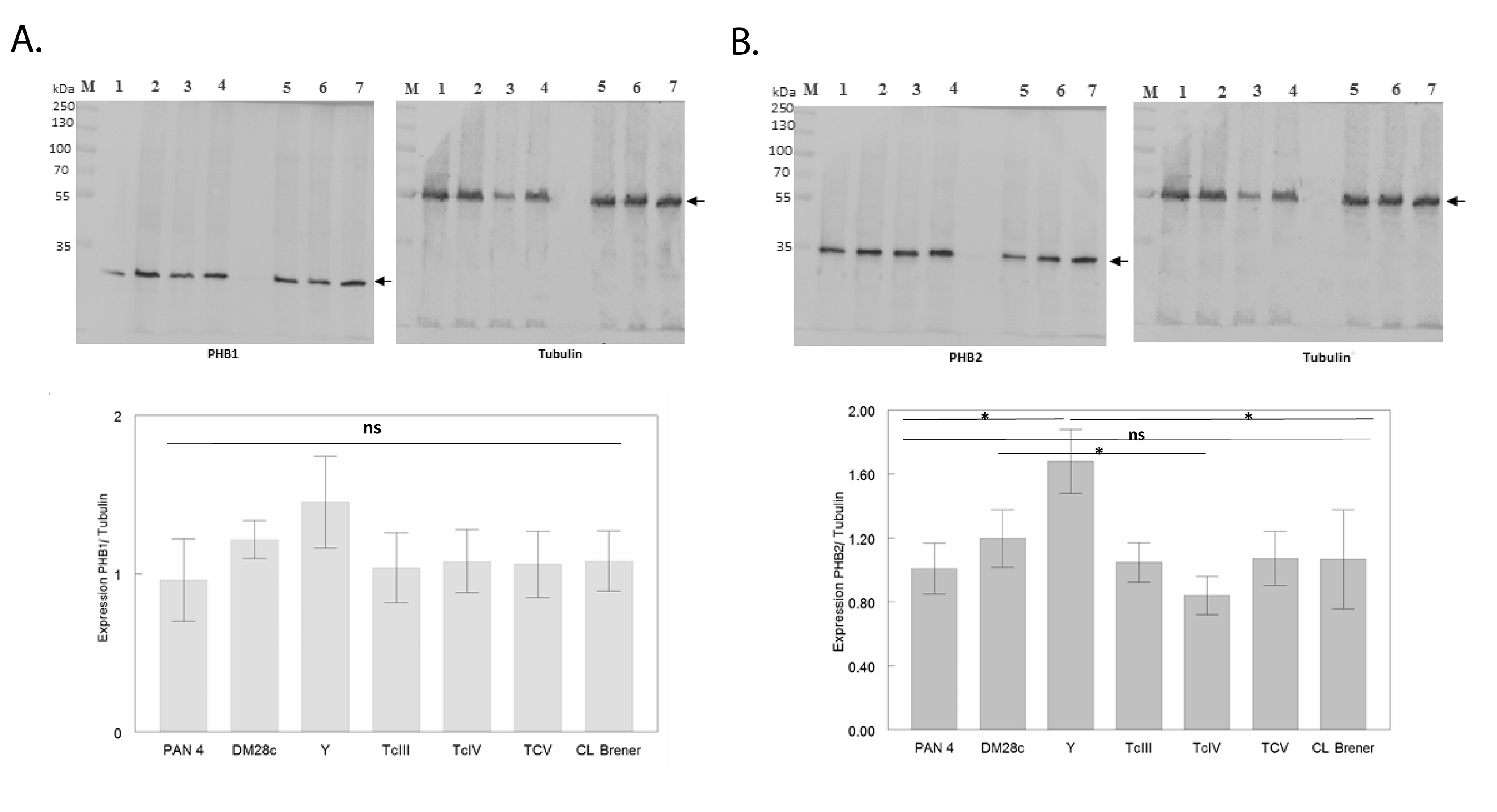

Supplement: S3 Fig — Relative expression of PHB1 (A) and PHB2 (B) versus tubulin in the epimastigote forms of T. cruzi classified in different DTUs. M: PageRuler prestained protein ladder (Thermo); total proteins of: 1: PAN4 strain (DTU I); 2: DM28c strain (DTU I); 3: Y strain (DTU II); 4: clone A3663 strain (DTU III), 5: clone 4167 strain (DTU IV); 6: MNCl2 strain (DTU V); 7: CL Brener strain (DTU VI). The ANOVA test did not reveal significant differences between the expression results of the parasites of different DTUs (p <0.05, ns: non-significant). (TIF) [file pntd.0009322.s004.tif]

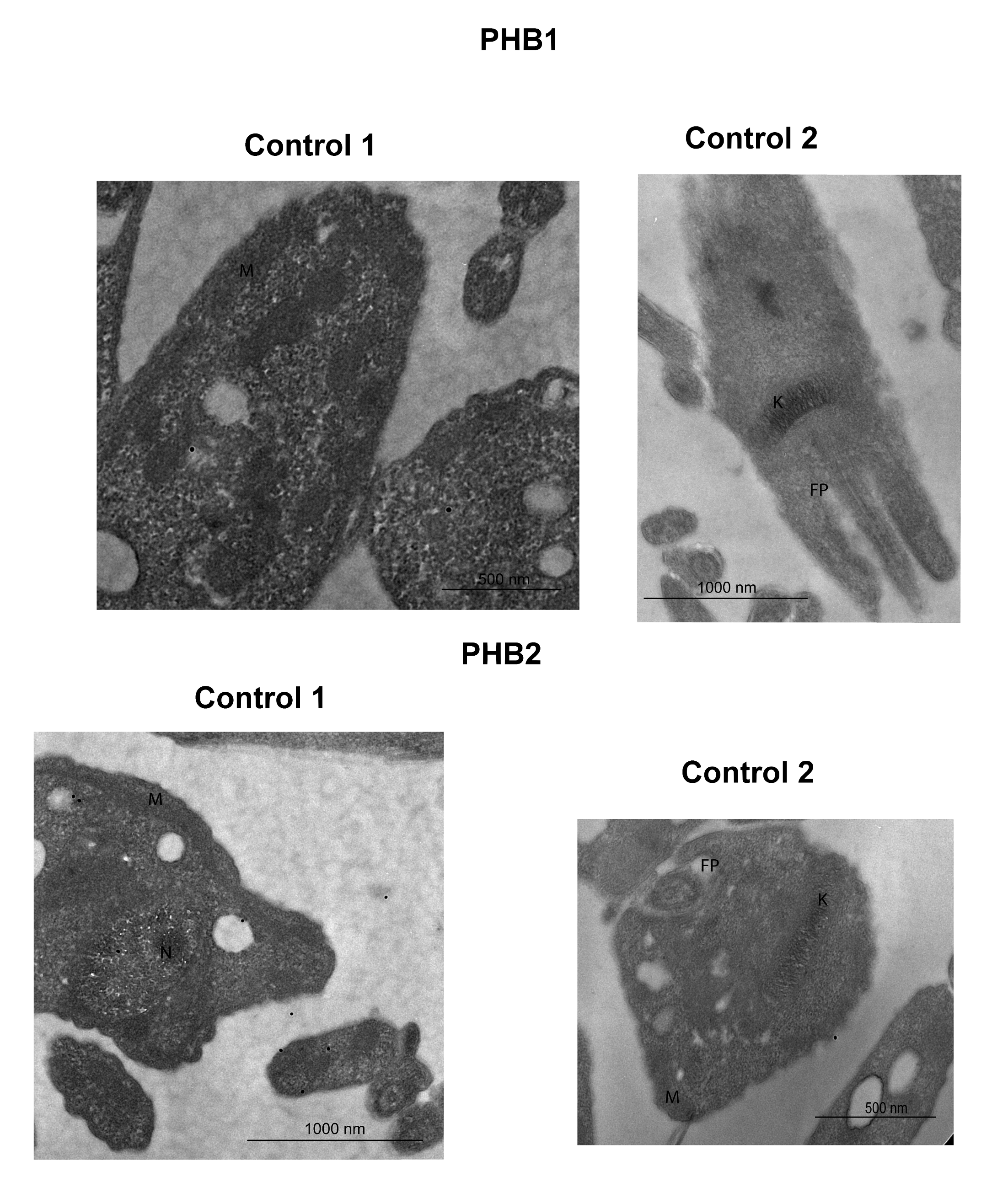

Supplement: S4 Fig — Control 1: parasites incubated with the preimmune serum and control 2: parasites incubated with serum from non-immunized animals. (TIF) [file pntd.0009322.s005.tif]

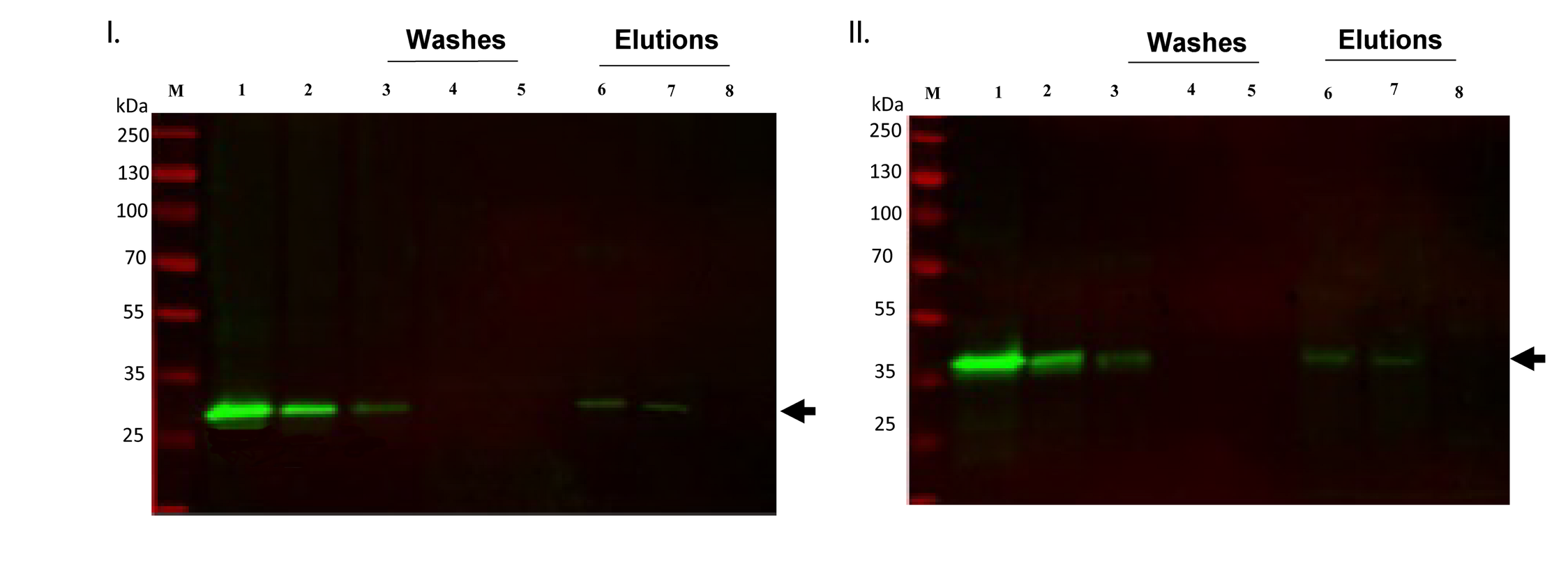

Supplement: S5 Fig — Detection of PHB1 (A) and PHB2 (B) after the affinity chromatography on Sepharose charged with Fe3+ ions by Western blot. M: PageRuler prestained protein ladder (Thermo); 1: total proteins in the epimastigote lysate of the DM28c strain; 2: total epimastigote proteins after the passage through the affinity column; 3: wash 1; 4: wash 2; 5: wash 3; 6: product of the first elution; 7: product of the second elution; 8: product of the third elution. (TIF) [file pntd.0009322.s006.tif]

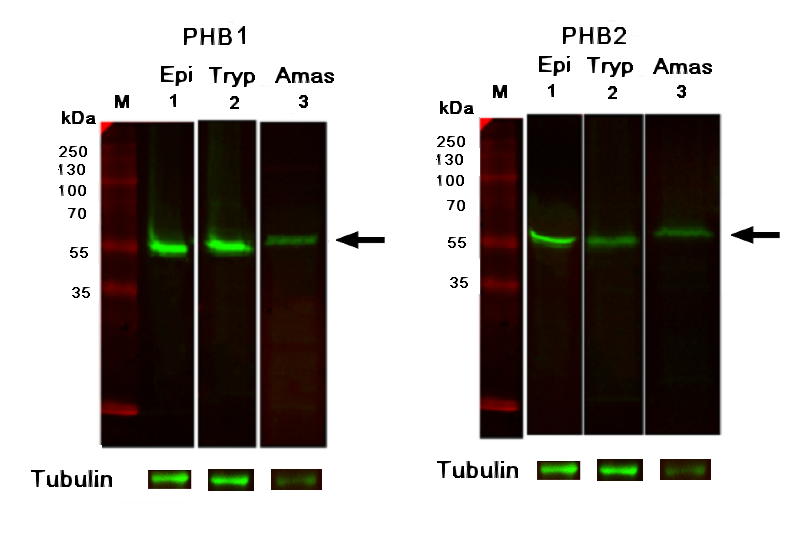

Supplement: S6 Fig — Overexpression of the recombinant PHB1 (A) and PHB2 (B) in T. cruzi epimastigote (1), trypomastigote (2) and amastigote forms (3), using an anti-CBP (Abcam) as primary antibody that recognizes the TAPtag. (TIF) [file pntd.0009322.s007.tif]

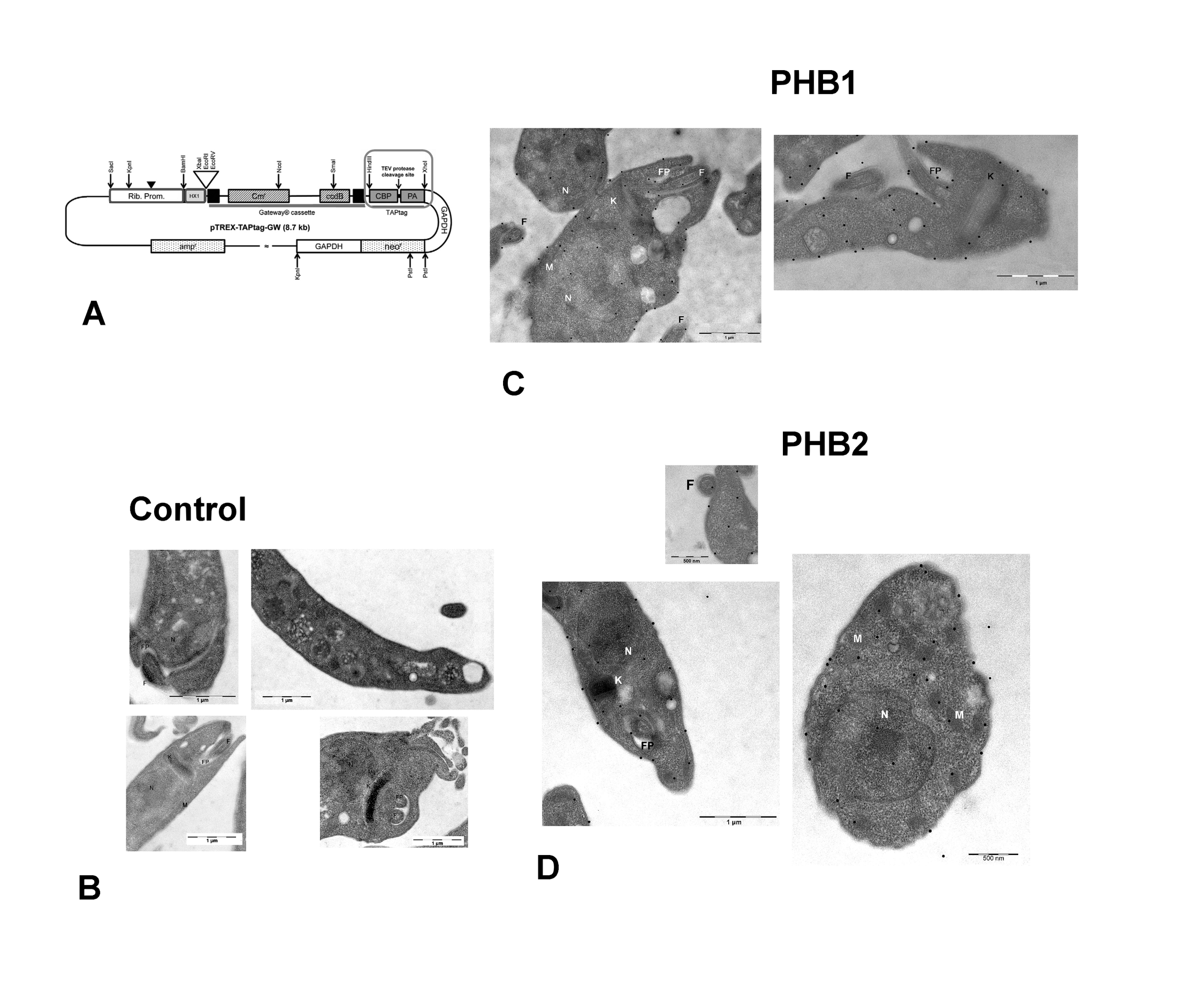

Supplement: S7 Fig — A. Circular map of the pTREX-TAPtag-GW vector: The GATEWAY (GW) cassette is shown in green and the TAPtag is shown in light blue, which consists of CBP (calmodulin-binding peptide), PA (protein A) and the cleavage site for TEV protease. The ribosomal promoter is seen in lilac and the HX1 region downstream in red. This plasmid possesses the resistance genes amp (ampicillin) and neo (neomycin). Flanking the neo gene are intergenic gapdh regions, according with [62,53]. B. Localization of PHB1 overexpressed in the epimastigote form and recognized by Protein A labeled with Au 20 nm. C. Localization of PHB2 overexpressed in the epimastigote form and recognized by Protein A labeled with Au 20 nm. (TIF) [file pntd.0009322.s008.tif]

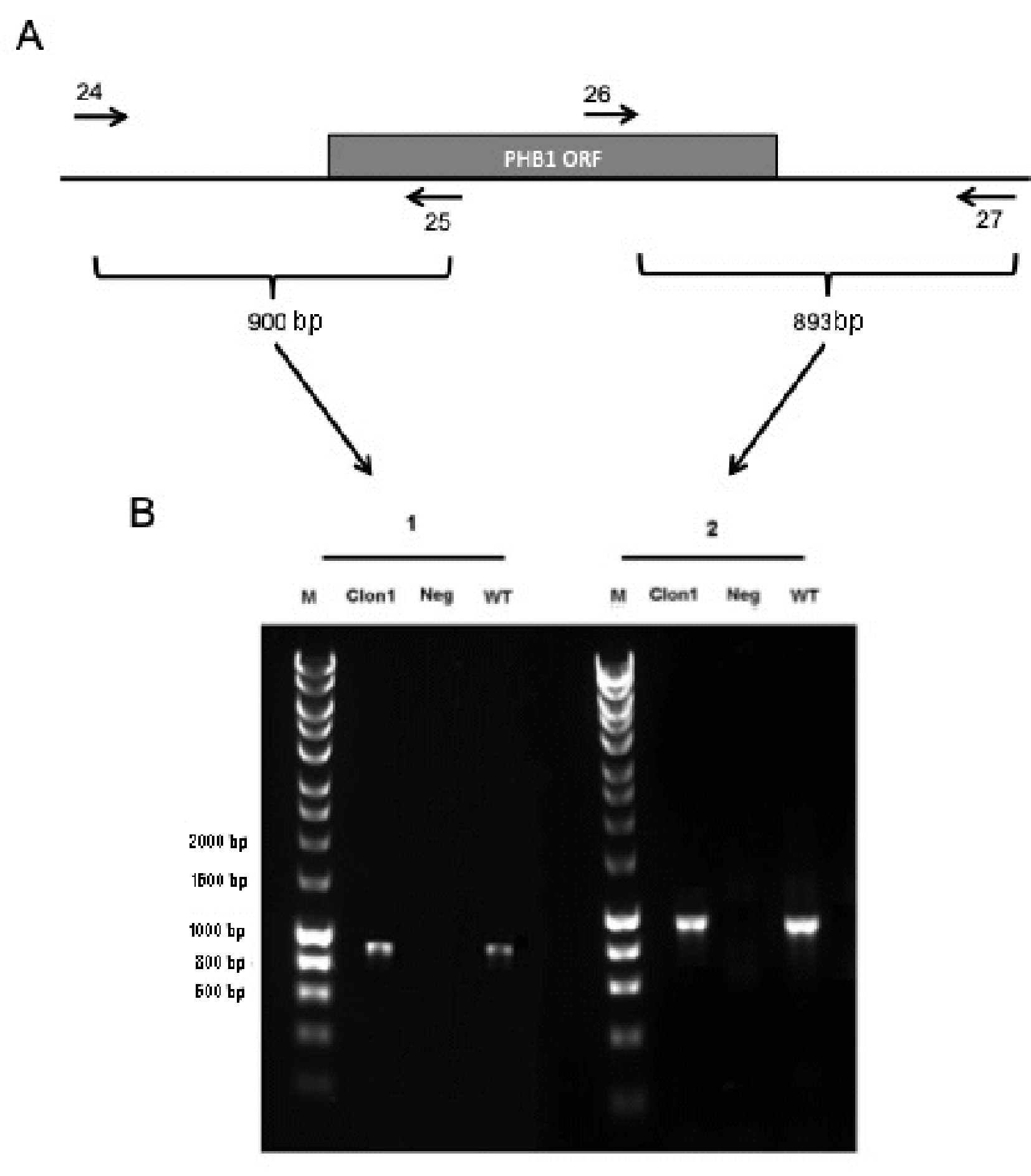

Supplement: S8 Fig — A. Schematic representation of the PCR strategy used to amplify the upstream and downstream regions of the PHB1 ORF. B. 1.5% Agarose gel showing the expected PCR products upstream (1) and downstream (2) the PHB1 locus in the PHB1 Clone 1 (CRISPR-Cas9 gene edited) and WT cell lines. (TIF) [file pntd.0009322.s009.tif]
